# WD-repeat instability and diversification of the *Podospora anserina hnwd *non-self recognition gene family

**DOI:** 10.1186/1471-2148-10-134

**Published:** 2010-05-06

**Authors:** Damien Chevanne, Sven J Saupe, Corinne Clavé, Mathieu Paoletti

**Affiliations:** 1 Laboratoire de Génétique Moléculaire des Champignons, IBGC, UMR 5095 Université Victor Segalen Bordeaux2 et CNRS, 1 rue Camille Saint-Saëns,33077 Bordeaux Cedex, France

## Abstract

**Background:**

Genes involved in non-self recognition and host defence are typically capable of rapid diversification and exploit specialized genetic mechanism to that end. Fungi display a non-self recognition phenomenon termed heterokaryon incompatibility that operates when cells of unlike genotype fuse and leads to the cell death of the fusion cell. In the fungus *Podospora anserina*, three genes controlling this allorecognition process *het-d, het-e *and *het-r *are paralogs belonging to the same *hnwd *gene family. HNWD proteins are STAND proteins (signal transduction NTPase with multiple domains) that display a WD-repeat domain controlling recognition specificity. Based on genomic sequence analysis of different *P. anserina *isolates, it was established that repeat regions of all members of the gene family are extremely polymorphic and undergoing concerted evolution arguing for frequent recombination within and between family members.

**Results:**

Herein, we directly analyzed the genetic instability and diversification of this allorecognition gene family. We have constituted a collection of 143 spontaneous mutants of the *het-R *(*HNWD2*) and *het-E *(*hnwd5*) genes with altered recognition specificities. The vast majority of the mutants present rearrangements in the repeat arrays with deletions, duplications and other modifications as well as creation of novel repeat unit variants.

**Conclusions:**

We investigate the extreme genetic instability of these genes and provide a direct illustration of the diversification strategy of this eukaryotic allorecognition gene family.

## Background

All living organisms have developed processes to discriminate self from non-self. Non-self recognition is required both in conspecific allorecognition (recognition of a different individual from the same species) or recognition of pathogenic non-self. In eukaryotes non-self recognition relies on the production of polymorphic proteins able to react to a range of molecules. Typically, genes involved in non-self recognition are among the most polymorphic loci in eukaryotic genomes, a classical example being the MHC/HLA loci in mammals [1]. Diversification of the corresponding genes occurs despite the exceedingly low overall mutation rate required to maintain genome integrity [2]. A challenge in evolutionary biology is to understand the mechanisms explaining this high level of polymorphism in non-self recognition genes. Clearly, positive selection for beneficial variants participates in the maintenance of polymorphism in non-self recognition genes, but in addition in a number of cases specific molecular mechanisms that increase diversification (and thus increase the panel of alleles on which selection can act on) are also involved.

In filamentous fungi conspecific non-self recognition happens in the heterokaryotic cell produced after the somatic fusion by anastomosis of cells of unlike genotypes [3-5]. The vegetative incompatibility (VI) process takes place when incompatible alleles of specific *het *loci are co-expressed in the cytoplasm of the heterokaryon and leads to a cell death reaction [6]. The biological function of VI and forces driving the evolution and maintenance of *het *genes remain unclear. VI is thought to restrict transmission of deleterious cytoplasmic elements such as viruses or abnormal mitochondria [7,8], and to limit resource plundering by aggressive genotypes [9]. It has also been proposed that genes controlling VI could in fact be involved in pathogen recognition and that VI could be a secondary consequence of the pathogen-driven divergence in these genes [10,11]. In that hypothesis, VI would be analogous to a genetic incompatibility phenomenon common in plants and known as hybrid necrosis. In hybrid necrosis a post-zygotic incompatibility occurs in F1 hybrids between different isolates and results from pathogen-driven divergence in genes with an immune function [12,13].

**Figure 1:**
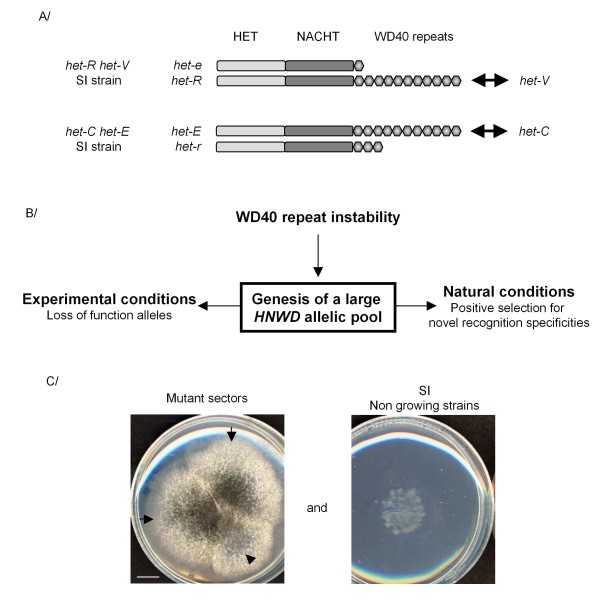
**Constitution of the RV collection of mutants**. A/Schematic representation of *het-e *and *het-r *alleles at the *hnwd *loci under study in the *het-R het-V *and the *het-C het-E *self incompatible strains. Plain arrowheads indicate non allelic incompatible interactions. B/Genesis and selection of new alleles at *hnwd *loci. During vegetative growth, the WD40 repeat instability promotes the rapid genesis of allelic variants at the *hnwd *loci for selection to act on. Our experimental set up selects for loss of function mutations while in natural conditions in the wild, selection will promote the maintenance of new recognition specificities. C/Selection of loss of function mutants in our experimental set up. Basically, SI strains were grown for 24 h in permissive condition before incubation for five days in non permissive conditions. Arrows indicate mutant sectors escaping from cell death from a single culture, as opposed to a culture where no mutant sector appeared. A single mutant per subculture was picked up for further analysis.

So far *het *loci have been characterized in only two species, *Podospora anserina *and *Neurospora crassa*. In *P. anserina *the *het-d *and *het-e *loci are involved in non-allelic incompatibility interactions with *het-c *(unrelated to *N. crassa het-c*) [14] while the *het-r *locus is involved in non-allelic incompatibility with *het-v *[15,16] (figure 1). Analysis of the recently sequenced *P. anserina *genome [17] revealed that *het-d*, *het-e *and *het-r *belong to the *hnwd *gene family that includes 2 additional members (*hnwd1 *and *hnwd3*). They are part of the larger *nwd *family that comprises five additional members [18]. *hnwd *genes encode for proteins including a N-terminal HET domain effector of cell death [19-21], a central NACHT domain [22] binding GTP and essential for activity [23], and a C-terminal WD repeat domain that varies in size (number of repeats) and sequence between natural isolates and defines allele specificity [24,25] (Figure 1). The five *hnwd *genes encode for proteins of the STAND class thought to act as molecular switches with a closed inactive form and an open active form adopted upon binding to an activating molecule [26,27]. The *nwd *genes display the NACHT and WD domains but lack the HET domain.

The WD repeat domains of the *P. anserina nwd *genes display very dynamic evolutionary features [18]. The WD domain is constituted of a variable number of highly conserved WD40 repeat units that are submitted to three evolutionary forces: 1) a high mutation load due to the high number of repeats and the possible action of RIP, a mutagenic process targeting repeated sequences [28], 2) concerted evolution resulting from WD40 unit sequence exchanges between and within loci and leading to WD40 repeat unit sequence homogenization, 3) strong positive diversifying selection acting on four positions encoding for amino acids located at the protein-protein interaction surface of the β propeller fold adopted by the WD repeat domains [18]. The combination of these evolutionary forces has the potential to constantly generate new alleles. This dynamism does not make obvious sense in the context of a role of these *het *genes restricted to VI. In contrast, in the context of the *hnwd *genes encoding for pathogen receptors contributing to an immune response [29,30], rapidly generating new alleles would allow for the surveillance of a wide range of pathogens.

It is well established that tandemly repeated sequences are unstable and undergo repeat number expansions and contractions. This repeat instability is at the origin of numerous cancers and neurological and developmental disorders in humans but in a number of cases this plasticity also appears advantageous as it can favour rapid genome evolution and adaptation [31,32]. Repeat variation rates in micro and minisatellite loci can be 5-orders of magnitude higher than rates of single-nucleotide substitution. Intragenic tandem repeat instability was for instance found to allow rapid evolution of cell surface antigens in fungi [33,34] but also of genes controlling morphological features in domestic dogs [35]. Mechanisms for this instability have been extensively studied and it turns out that any mechanism involving new DNA synthesis such as replication, recombination and repair can contribute to repeat rearrangements [32].

In the present paper, we took advantage of the genetic tractability of non allelic VI systems of *P. anserina *to directly analyze rearrangements in intragenic tandem repeats in this eukaryotic non-self recognition gene family. We selected a collection of 143 loss of function specificity variants in two members of the *hnwd *family spontaneously generated under vegetative growth conditions (figure 1), while in nature selection would maintain alleles providing new recognition specificities. We then analyzed the underlying molecular events that occurred. We found that diversification of the WD-repeat domains occurs through intragenic reshuffling in the repeat arrays. Importantly, this reshuffling is accompanied by frequent generation of novel repeat unit sequences. The consequences of this diversification in the context of non self recognition in natural isolates will be discussed. This study provides a direct illustration of the role of tandem repeat instability in evolvability in the context of non-self recognition.

## Results

### Mutational bias towards *hnwd *genes

In non-allelic incompatibility systems, incompatibility is expressed upon cell fusion between incompatible strains but also in the progeny of sexual crosses involving incompatible parental strains. Incompatible gene pairs (such as *het-R het-V *or *het-C het-E) *can be associated in a single haploid nucleus by appropriate genetic crosses, leading to the production of so-called self incompatible (SI) strains (Figure 1). It has long been described that spontaneous mutants escaping cell death arise from SI strains [16]. The vast majority of the mutations allowing escape from *het-R het-V *or *het-C het-E *incompatibility occur in the *het-R *or *het-E *genes respectively while inactivation of *het-V *and *het-C *is rare. The underlying cause for this differential "mutability" was not understood. We generated two collections of spontaneous mutants allowing escape from *het-R het-V *or *het-C het-E *incompatibility, the different steps of the process being presented figure 1 and in Materials and Methods. Basically *het-R het-V *and *het-C het-E *SI strains were grown for 24 h in permissive conditions suppressing the manifestations of VI before transfer to non permissive conditions where the incompatibility reaction proceeds. After 5 days, 299 spontaneous mutant sectors recovering normal growth arose from which we picked and analysed a single mutant per subculture to generate the RV and CE spontaneous mutant collections. The RV and CE collections include respectively 109 and 34 unique individuals.

Mutations allowing for suppression of incompatibility could occur *a priori *in either one of the *het *genes composing the gene pair causing the incompatible interaction of the SI strain (*het-R het-V *and *het-C het-E)*, or in extragenic suppressor which we did not observe. For each collection, we identified the mutated *het *gene by confronting each mutant against both parents used to set up the original cross. Incompatibility is characterized by the formation of an abnormal contact zone between incompatible strains called the barrage [6]. For each gene pair, the mutations were totally biased towards one of the two *het *genes. All the mutants of the RV collection were affected at the *het-r *locus and expressed an inactive *het-r *allele. As well, all the mutants of the CE collection were affected at the *het-e *locus and expressed an inactive *het-e *allele. The combined 143 mutants from both collections are affected in the *het *genes that belong to the *hnwd *gene family suggesting a particular genetic instability at the *hnwd *loci.

### Repeat loss is the most common event for *hnwd *inactivation

**Figure 2:**
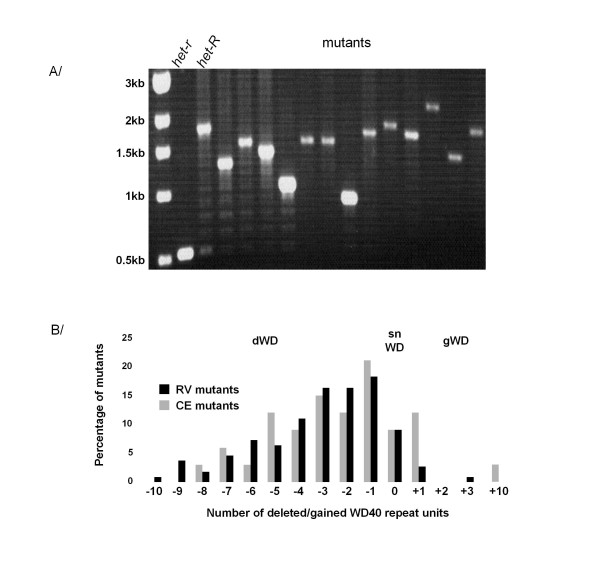
**Classes of mutants identified by the number of WD40 repeat units**. A/The WD domain of the *het-r *gene of a selection of mutants from the RV collection were PCR amplified and separated on an 0.8% agarose gel (molecular marker: 1 kb ladder). The sizes of the amplicons are compared to the natural inactive (*het-r) *and active (*het-R*) alleles. B/Three classes of mutants are defined according to the size of the WD repeat domain of the *het-r *or the *het-e *gene: the deletion mutants (dWD), the gain of WD40 repeat mutants (gWD) and the mutants with the same number of WD40 repeats as the initial allele (snWD). Subclasses of mutants are defined according to the number of WD40 repeat units deleted (-) or gained (+) compared to the initial allele. The percentage of the total number of mutants comprised in each subclass is represented.

We set out to define the molecular events leading to inactivation of the *hnwd *genes. The presence of highly conserved tandemly repeated WD40 sequences both in the *het-E *and *het-R *genes led us to investigate the status of the repeat domains. The number of WD40 repeat units present in the WD repeat domains of the mutated genes (*het-r *or *het-e*) was estimated from the size of the PCR amplicon generated using locus specific primers (additional file 1). The initial active *het-R *allele and the *het-E1 *allele used here both contain 11 WD40 repeats [15]. On the basis of the number of repeats in the WD domain of the mutants, we defined three classes of mutants, the class of deletion mutants (dWD) that present a reduced number of WD40 repeats compared to the wild type alleles, the class of gain of repeat mutants (gWD) that have a greater number of WD40 repeat units than the wild type allele, and the class of mutants with the same number of WD40 repeats as the initial active allele (snWD). As revealed in figure 2, for both collections most mutants belong to the dWD class as 87% (95/109) of the RV mutants and 79% (27/34) of the CE mutants have less than the original 11 WD40 repeats. In addition we found 10 snWD class and 4 gWD class mutants in the RV collection, and 3 snWD class and 4 gWD class mutants in the CE collection. The proportion of dWD mutants was to be expected as it was shown that a minimum of ten WD40 repeat units are required for VI to occur [24].

### Mapping of the WD40 repeat deletions

**Figure 3:**
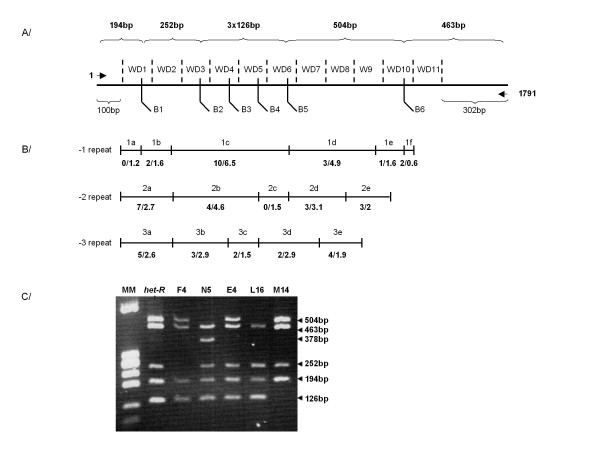
**Mapping of the WD40 repeat deletions**. A/On this schematic representation of the *het-R *WD repeat domain dashed lines delineate the WD40 repeat sequences and *Bgl*II restriction sites are labelled B1 to B6. At the top are indicated the size of the expected *Bgl*II generated restriction fragments and below are indicated the size of the non repeat fragments from the end of the amplicon to the beginning of the repeated sequences. B/For the three most represented subclasses of mutants, the intervals in which deletion occur are represented. Top labels refer to the intervals defined (additional file 2). Numbers below each interval represent the number of observed vs the number of expected mutants is generated at random. C/Representative agarose gel presenting the *Bgl*II restriction profiles of the wild type *het-R *allele along with selected mutants. F4, N5 and E4 belong to the -1 subclass, L16 to the -2 subclass and M14 to the -3 subclass.

Most mutants are thus generated through deletion of a number of WD40 repeat sequences. Deletions of a small number of WD40 repeats are more frequent than deletions of a large number of repeats (figure 2). We took advantage of the presence of *Bgl*II restriction site polymorphism in the WD40 repeat sequences of the *het-R *parental allele [15] to map the deletion events of the RV deletion mutants collection (Figure 3). First we predicted all possible patterns generated by *Bgl*II restriction of PCR fragments amplified with primers A and B from genomic DNA of the *het-R *wild type allele or all the deletion mutants of the RV collection (additional file 2). Then, assuming that only deletions of contiguous WD40 repeats occurred, we predicted the intervals in which deletions could occur to generate the predicted *Bgl*II restriction profiles (additional file 2). The fact that 88 out of 95 mutants resulted in predicted *Bgl*II restriction profiles suggests that indeed deletions of a unique fragment of contiguous WD40 repeat sequences is the most frequent event. The 7 mutants not producing predicted *Bgl*II restriction profiles probably resulted from more complex rearrangements. Finally for the 3 most frequent subclasses of mutants as defined by the number of deleted WD40 repeats, we compared the number of occurrence of deletions in each interval to (E), the number of excepted deletions per interval if they occurred at random along the sequence. E depends on the length of the interval, the length of the WD repeat domain and the number of mutants in the subclass (Figure. 3 and additional file 2). This analysis demonstrated that deletions can involve any of the 11 repeats and that deletions occur at random along the WD40 repeat domain. We could not conduct the same analysis for the *het-e *mutants as *Bgl*II sites were present in 10 of the 11 WD40 repeats.

### Sequence of 25 mutant alleles

To characterize the molecular events leading to inactivation of the *het-R *or *het-E *alleles in the different classes, we systematically sequenced the WD domains from the 21 individuals of the snWD and gWD classes of mutants as well as 4 alleles of the dWD class selected randomly. Five of the snWD mutants from the RV collection display a WD repeat domain identical to wild-type but displayed mutations outside of the WD-domain and will be described in a further section.

**Figure 4:**
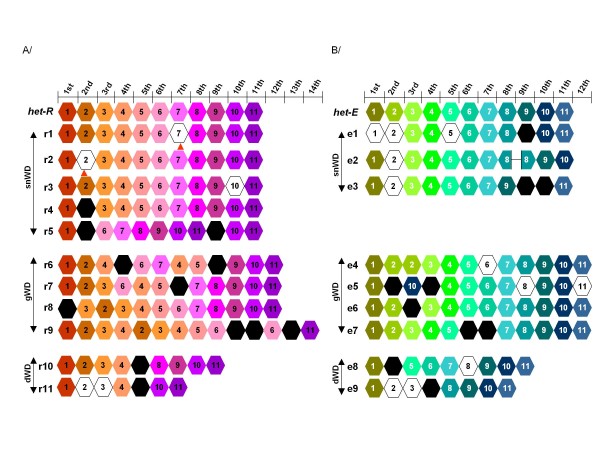
**Schematic representation of the gWD and snWD mutants**. A/Parental active *het-R *allele and mutants from the RV collection. B/Parental active *het-E *allele and mutants from the CE collection. Nucleic sequence WD40 repeat units sharing the same colour are identical. Repeat units represented in white differ by a single nucleotide from their parental repeat unit (indicated by a number). Repeat units represented in black differ by more than one nucleotide from any parental units. Stop codons are indicated by arrowheads. Incomplete boxes of the e2 mutant delineate a 21 bp in frame deletion.

The remaining 16 alleles were affected in the WD-repeat domain (figure 4). Overall we found 38 new WD40 repeat sequences as compared to the parental alleles. First mutations in the WD repeat domain all preserved the reading frame. In the simplest situation mutant alleles differed from wild type by a single point mutation in one of the WD-repeat units as exemplified by the *r1 *and *r2 *alleles mutated in repeat unit 7 and 2 respectively. Then, in the sequenced dWD alleles, a single deletion step led to the removal of 2, 3 or 4 contiguous repeat units in the *r10*, *r11, e8 *or *e9 *deletion mutants. Duplication of one or several repeat units is also observed like duplication of repeat unit 2 in the *e4 *allele. Complex rearrangements occurred in several alleles with concomitant duplication, replacement and deletion events that imply multiple steps as exemplified by allele *r9 *which displays a total of 14 repeat units.

### Repeat rearrangements are intragenic

One of the striking features of the mutant allele sequences is that in the vast majority of the alleles, one finds new WD repeat units. We found 16 repeat units that differ from a parental unit by a single mutation and 22 repeat units that differ from the parental units by more than a single point mutation (figure 4). We observed 12 such new WD40 repeat unit sequences in the RV mutant collection and 10 in the CE mutant collection (figure 4). To define the origin of these 22 sequences, and because the WD40 repeat sequences of the *hnwd *gene family are undergoing concerted evolution and can thus occasionally be exchanged between loci, we sequenced the WD domain of all the family members from both parental strains used to produce the *het-R het-V *and *het-C het-E *SI strains. This lead to the identification of 41 new WD40 repeat units in addition to the 69 units already found in the sequenced genome [36]. A high level of polymorphism is evident at the *nwd *loci between the two parental strains, both in terms of number of WD40 repeat units present at each locus and in terms of WD40 repeat unit sequences (additional file 3). As these strains are nearly isogenic [37], the observed polymorphism could reflect polymorphism existing in the natural isolates from which they are derived. Alternatively, this polymorphism could be the result of unselected concerted evolution occurring during crosses realised in laboratory conditions over the years as observed for rDNA loci in *Drosophila melanogaster *[38] or *Daphnia obtus *[39]. A phylogenetic tree was then constructed with the nucleotide sequences of the 157 WD40 repeat units from the wild-type alleles and including the 29 new WD40 repeat units identified in the mutant *het-r *and *het-e *alleles (additional file 4) that revealed a topology consistent with what we previously observed [18]. The WD40 repeat units tend to cluster by gene of origin with a strong bootstrap support. This indicates that intragenic events leading to sequence homogenization of WD40 repeat sequences are more frequent than intergenic exchanges. Significantly, all the newly identified WD40 repeat sequences found in the *het-r *mutant alleles cluster with the wild-type WD40 repeat sequences from the *het-R *allele. Similarly, all newly identified WD40 repeat sequences at the *het-e *locus cluster with the WD40 repeat sequences from the parental *het-E *allele. We also analyzed the WD-repeat number of all *nwd *gene family members in all mutant strains. None of the other *nwd *genes showed any variation in repeat unit number in the mutants, consistent with the conclusion that rearrangements occurred at the intragenic level only (data not shown).

From these observations, we conclude that although intergenic WD40 repeat unit exchanges between *nwd *loci apparently occur in the wild [18], in our experiment the new WD40 repeat sequences have been generated through intragenic events only.

### Rearrangements generate multiple novel repeat unit sequences

As mentioned above in most of the mutant alleles one or several repeat units with novel sequences are found. One can attempt to recapitulate the events that led to the genesis of such novel repeat units. The simplest situation is probably the one that occurred in the alleles of the dWD class. Mutants *r10 *and *r11 *of the RV collection were deleted of 2 or 4 WD40 repeat units respectively. The deletion event resulted from fusions between repeats 5 and 7 and between repeats 5 and 9 of the wild-type allele in mutants *r10 *and *r11 *respectively. This fusion event generates new WD40 repeat units sequences. The same was true for the two dWD mutants of the CE collection (*e8 *and *e9*). Again we found that in both cases a single fragment was deleted and resulted in new chimeric WD40 repeat sequences (additional file 5).

In mutant *r4 *allele, 7 point mutations clustered in 88 bp are observed in the 2^nd ^repeat of the domain. This stretch of 88 nucleotides appears almost identical to the corresponding region of the 4^th ^WD40 repeat sequence of the wild type allele and differs by only two positions located at the end of this region (additional file 6). This cluster of mutations could thus be the result of gene conversion of this short region in the mutant WD40 repeat sequence by the wild type sequence of the 4^th ^WD40 repeat unit.

A simple case of unequal crossing over could explain the genesis of the mutant allele e6. This mutant has gained a WD40 repeat unit e6-3 whose sequence is new. Comparison of the WD40 unit sequences reveals that this new repeat could correspond to a fusion between the 5' end of the third repeat and the 3'end of the second repeat of the wild type allele. The break point could have occurred at the adenine nucleotide at position 49 that is unique amongst the WD repeats (additional file 7).

We have previously shown that two pairs of codons (pairs of codons 7, 9 and 25, 27) in each repeat unit are under positive selection. Based on homology modelling, the corresponding amino acids all group at surface loops at the top of the β-propeller structure at the interaction surface [18]. The amino acid combination at those four positions thus presumably defines the functional binding properties of the repeat unit. In the 41 new WD40 repeat units we identified in the whole of the *nwd *gene family, 23 displayed new amino acids and associations of amino acids at these four positions that had not been described in the genome sequence (additional file 8). More significantly, among the 38 new WD40 repeat units generated through mutations of the *het-E *or *het-R *alleles, twelve display novel amino acid combinations at those 4 critical positions that were not found in the repeat units of the parental alleles (table 1). Nine of these new combinations apparently arose by shuffling parental pairs of codons. However three mutant WD40 sequences most likely generated by point mutations lead to pairs of codons absent from the parental sequence. This observation strongly suggests that repeats with novel functional properties can indeed be generated through these rearrangement events as demonstrated in vitro for TPR or LRR containing repeat units [40,41].

**Table 1:** Amino acid combinations at the protein protein interaction surface.

**A/**					**B/**				
	**Position**					**Position**			
			
	**7**	**9**	**25**	**27**		**7**	**9**	**25**	**27**
	
e1-1(snWD)	S	L	**N**	**K**	r10-5(dWD)	S	S	G	D
e1-9(snWD)	W	H	D	H	r11-2(dWD)	S	Y	D	D
e3-2(snWD)	**T**	**W**	D	K	r11-3(dWD)	S	S	G	D
e5-2(gWD)	S	W	S	N	r11-5(dWD)	S	S	G	D
e5-4(gWD)	S	W	D	H	het-R-1	S	Y	G	R
e6-3(gWD)	R	Q	D	K	het-R-2	S	Y	V	D
e7-7(gWD)	W	Q	D	K	het-R-3	S	Y	G	D
e9-5(dWD	S	W	**I**	**D**	het-R-4	S	S	V	R
het-E-1	S	L	D	K	het-R-5	S	S	V	D
het-E-2	S	W	D	K	het-R-6	S	S	G	R
het-E-3	R	Q	D	H	het-R-7	W	Y	G	D
het-E-4	S	L	G	K	het-R-8	S	S	D	D
het-E-5	S	W	G	K	het-R-9	L	Y	G	D
het-E-6	S	W	D	K	het-R-10	S	H	V	D
het-E-7	W	Q	D	H	het-R-11	S	S	V	C
het-E-8	S	W	I	G					
het-E-9	W	H	I	G					
het-E-10	W	Q	S	K					
het-E-11	W	Q	S	N					

The four amino acids at positions under selection in newly generated alleles (underlined) are amino acids found in the parental sequences (white boxes) for A/the *het-E *gene, and B/the *het-R *gene. Bolded pairs of amino acids were generated in the mutants. Repeats are named after their positions in the mutated alleles, the class of the corresponding mutant is indicated in brackets.

### Point mutations outside of the WD-repeat domain

Five of the snWD mutants from the RV collection display a WD repeat domain identical to the wild type repeat domain. We then sequenced the remaining of these mutant genes after PCR amplification with primers C and D (additional file 1). After comparisons with the wild type allele we identified one nucleotide insertion in mutants E5 and M8 and a one nucleotide deletion in mutant A1 that lead to frame shifts and premature stop codons (additional file 9). In the J4 mutant we identified a T66G substitution that results in an I22 M mutation (additional file 9). Interestingly this position belongs to the first of three conserved blocks forming the HET domain as defined earlier [19] and responsible for the induction of the cell death reaction [21]. In the M12 mutant a T1702A substitution leads to a Y568N mutation (additional file 9). This position is located between the conserved block V and VI of the NACHT domain [22] that is present in many different proteins of the STAND class [26]. The NACHT domain activity is essential to the incompatibility reaction [23].

## Discussion

We have selected for mutations in two members of the *hnwd *gene family that suppress self-incompatibility. In other words the incompatible genetic interaction between the *HNWD *genes and their antagonist *het-C *or *het-V *genes has been lost through mutation of the *HNWD *partner. The vast majority of mutants were altered in their WD repeat domain involved in recognition specificity. The WD repeat domain adopts a doughnut shaped β propeller fold that forms a protein-protein interaction surface at its top [42]. A typical β propeller fold associates seven WD40 repeats, although such structures containing between 4 to 8 repeats have been described [43,44]. Proteins including larger number of WD40 repeat units have been described, and for example the thirteen WD40 repeat units of APAF-1 forms two distinct β propeller structures of six and seven repeat units [45,46]. As we do not know how the WD repeat units of the HNWD proteins assemble, the reasons for the loss of activity in the mutants can only be speculated, but it is reasonable to suspect that loss of the genetic incompatibility in the mutants results from alterations of the physical interaction between the WD repeat domain of HET-R or HET-E proteins with their antagonist partner HET-V or HET-C. Most mutations correspond to deletions of WD40 repeat units. Large deletions leading to WD repeat domains encoding for less than 6-7 WD repeat units are unlikely to associate into a functional β propeller thereby producing an inactive protein. In contrast small deletion mutants, snWD mutants and gWD mutants retain a number of repeats sufficient to generate a β-propeller fold. This suggests that the mutations specifically affect the binding affinity of the WD domain for the antagonist partner after the rearrangement of the amino acids located at the protein-protein interaction surface. The fact that in the RV collection repeat deletions that inactivate *het-R *occur all along the sequence of the repeat domain suggests that all the WD40 repeat units contribute to binding of the antagonist partner.

We obtained a total of 299 spontaneous mutants sectors starting from 153 colonies. All the mutants analysed are affected in the *hnwd *gene involved in the system. We found no mutants affected in the *het-C *or *het-V *genes, or in extragenic suppressors. Also interestingly in a similar screen for mutants escaping from VI cell death with a genetically engineered *het-s*/*het-S *SI strain, Coustou and co-authors obtained only 17 mutants from 500 starting colonies after mutagenic treatment [47]. This difference in the mutant screening yields highlights the instability of the *hnwd *genes as opposed to the repeat-free *het-s*, *het-S *genes, *het-c *or *modA *genes [48]. In that respect it is relevant to note that the *het-E *allele we used here displays an additional WD40 repeat unit compared to the allele originally described [25] while both recognize the same *het-C1 *allele. This difference is the likely consequence of genetic modification that occurred during subcultures in laboratory conditions.

A remarkable consequence of the instability of the WD repeat domain is that it contributes to the diversification of the WD40 repeat repertoire. Not only are repeat arrays reshuffled and specific repeats duplicated or deleted but numerous novel repeat unit sequences were generated in the process. In the subset of mutants we analysed we found a total of 38 new WD40 repeat units. More significantly, we found 12 new combinations of the four positions of the WD40 repeat unit located at the top of the β-propeller structure and thought to be crucial for recognition of the antagonistic partner [18]. We used here an experimental set up starting from a SI strain grown under permissive conditions and transferred to non permissive conditions to select for loss of function mutations as a consequence of the plasticity of the WD repeat domain of the *hnwd *gene family (figure 1). The genetic plasticity unveiled here might provide an explanation for a remarkable result obtained in early genetic studies on incompatibility interaction in *P. anserina*. Delettre and Bernet [49] have shown that using an *ad hoc *screen several additional non-allelic incompatibility loci could be genetically selected in laboratory conditions. The incompatibility interactions selected in that manner shared a number of features with the *het-C/het-E *and *het-R/het-V *interactions, for instance they could be alleviated by the suppressor mutations that suppress *het-C/het-E *and *het-R/het-V *incompatibility. These new *het *loci also appeared to be genetically unstable. Based on the results presented herein, a plausible explanation for this result is that rearrangements in repeat arrays of *hnwd *genes generated novel binding specificities leading to recognition of a self determinant creating a genetic incompatibility *de novo*. Similarly, in nature, from the pool of WD40 repeats arrangements produced in the mycelium under vegetative growth conditions, selection will fish out combinations providing novel advantageous recognition specificities. It is relevant to note that *het-c*, the antagonistic locus of *het-d *and *het-e *in VI, also displays signs of fast evolution [29] suggesting a co-evolution with the *hnwd *genes, thus new binding specificities might also be generated through evolution of *het-c*.

In biotechnological applications, it has been demonstrated *in vitro *that random association of different ankyrin repeat units can produce engineered ankyrin domains with binding affinity for selected targets [40,41], the same is true for designed LRR- and tetratricopeptide-repeat proteins. The combinatorial reshuffling and individual repeat generation process unveiled here suggest that the WD40 domains of *nwd *genes might allow generation of a virtually unlimited binding repertoire. The generation of extensive binding repertoires by combinatorial association of variable intragenic tandem repeats is a strategy that is also encountered in other eukaryotic non-self recognition molecules in plants and animals. In plants recognition of pathogens is ensured by members of the *NBS-LRR *genes encoding for a NBS nucleotide binding site and a Leucine Rich Repeat domain. The LRR domain is thought to contribute to pathogen recognition [50,51]. Some *LRR *genes are submitted to positive diversifying selection, concerted evolution and recombination between and within loci [52,53]. Evolution of the *NBS-LRR *genes appears enhanced in presence of the pathogen as presence of pathogen attack induces the Systemic Acquired Recombination [54] preferentially in the *NBS LRR *encoding genes [55]. Similarly, in jawless vertebrates antigen recognition by lymphocyte receptors relies on the combinatorial assembly of LRR gene segments [56]. It has been proposed that somatic recombination in the variable LRR-units has the potential to generate a repertoire of 10^14 ^different lymphocyte receptors. It appears that diversification of the fungal *hnwd *non-self recognition genes follows similar principles including diversification and combinatorial shuffling of repeated units forming a recognition plateform. The estimated WD-domain repertoire is colossal (>10^12^) even if one only considers the 54 different repeat units identified in the sequenced genome as the building blocks for a 7 repeat WD-domain repertoire. This likely represents an underestimation since additional repeat unit variants were found in a different isolate and novel repeat units can be generated through repeat instability. In addition, the β-propeller structures might be made of more than 7 repeats as exemplified by the 11 repeat WD-domains of wild-type *het-e *and *het-v*[48].

In the two examples cited above, the plant *NBS-LRR *genes and the sea lamprey lymphocyte receptors, the repeat proteins are involved in pathogen-detection and the extraordinary binding versatility of these proteins is related to the diversity of the pathogen associated patterns that are recognized. Now how should the evolvability of the *hnwd *genes be understand? The possibility to generate such a vast variety of binding specificities does not make obvious sense in the context of vegetative incompatibility especially considering that in *N. crassa*, incompatibility alleles are very stable, in finite number (two or three alleles) and evolutionarily very ancient. The evolvability of the *hnwd *genes along with other features of the VI reaction recently lead us to suggest that the *hnwd *genes might be components of a fungal innate immune system with the function in response to pathogenic non-self [11]. In this context, as in the two examples mentioned above in plants and jawless vertebrates, the ability to produce a wide range of non-self recognition molecules would allow for the detection of a wide range of pathogens and counteract their immune-avoidance strategies. It is interesting to note that in some cases cell surface antigen in pathogens rely on tandem repeat instability for rapid adaptation so it appears that similar weapons are use by host and pathogens in their ongoing arms race.

## Conclusions

The causes and the consequences of genetic incompatibilities currently raise a keen interest among evolutionary biologist because of their role in speciation processes [12,57-59]. Fungal incompatibility provides a genetically well defined example of such a genetic incompatibility. *hnwd *genes based non-allelic incompatibility in *P. anserina *leads to various degrees of pre and post-zygotic isolation [60]. The present study documenting the genetic instability and plasticity of the *hnwd *genes as well as the hybrid necrosis phenomenon caused by *NBS-LRR *genes in *A. thaliana *[12,13] illustrate that particular evolutionary regimens promoting extensive and rapid variation among isolates of the same species can favour the arising of genetic incompatibilities.

## Methods

### Strains, incompatibility relationships and selection of mutants

Conditions and media used for *P. anserina *have been reviewed recently [61]. The reference *P. anserina *isolate called *s *harbours the *het-r*, *het-V*, *het-c2 *and *het-e4 *alleles at the *het-r*, *het-v*, *het-c *and *het-e *loci and is nearly isogenic to all other strains used in this work [37]. Because these loci are involved in non allelic incompatibility, it is possible to recover self incompatible (SI) strains in the progeny of appropriate crosses. SI strains display a clear phenotype characterised by a growth arrest soon after spore germination and a generalized cell death reaction. A cross between the *s *strain and a strain bearing the *het-R *and *het-V1 *alleles at the *het-r *and *het-v *loci will produce SI *het-R het-V *F1 progeny. As incompatibility between *het-R *and *het-V *alleles is thermosensitive, these SI strains can grow as wild type at 32°C, while transfer to 26°C triggers the incompatibility reaction [16]. A cross between the *s *strain and a strain bearing a single incompatible allele at the *het-e *locus (*het-E*_*a*_) produce a *het-C het-E *SI strain. Incompatibility in these SI strains is partially suppressed by the addition of 6 g/l of dihydrostreptomycin in the culture medium [62]. We selected and grew *het-R het-V *or *het-C2 het-E *SI strains in permissive conditions (32°C or dihydrostreptomycin containing medium) for 24 h until the mycelia reached 1 cm in diameter, before transfer to non permissive conditions (26°C or dihydrostreptomycin free medium). After 5 days of incubation we observed spontaneous mutant sectors arising. We collected a single mutant sector per starting colony to avoid analysing several times the same mutant. Vegetative incompatibility was assessed by barrage testing and sexual compatibility was assessed by spermatization as previously described [15].

### Nucleic acid manipulation and nomenclature

Routine genomic DNA extractions were performed as described by [63], or using the Plant DNA extraction kit (Qiagen) when high quality DNA was necessary. PCR were performed using the Long Range Template Taq polymerase (Roche), according to the manufacturer recommendations. Time for DNA extension was adjusted according to the length of the fragment to amplify. The oligonucleotides used, as well as their location in the various loci analysed are listed in additional file 1. PCR products were gel purified and cloned in the XL TOPO cloning vector (Invitrogen). Sequencing was performed by the Genome express company. We systematically sequenced cloned WD40 sequences from at least two independent PCR reactions. Accession numbers are listed in additional file 10. Nucleic acids and protein sequences were analysed with the program MEGA3 [64]. PCR fragments were column purified (Qiagen Minelute kit) before restriction with *Bgl*II (Biolabs).

Gene and loci names are italicized while protein names are not. Active alleles are indicated with capitals while inactive alleles and loci are indicated with lower cases.

## Authors' contributions

DC and MP carried out the experimental work. CC, SJS and MP participated in the design of the study and drafted the manuscript. All authors read and approved the final manuscript.

## Supplementary Material

Additional file 1 Details of the primers used. Click here for file

Additional file 2 Mapping of the deletions occurring in the dWD class of mutants of the RV collection. Click here for file

Additional file 3 Schematic representation of the parental WD repeat domains of the *hnwd *gene family members. Click here for file

Additional file 4 Neighbor joining phylogenetic tree constructed with the nucleic sequences corresponding to WD40 repeat units from all members of the *P. anserina nwd *gene family, along with the new complex WD40 repeat unit sequences from the RV and CE collections. Click here for file

Additional file 5 Protein sequences of WD repeat domains of randomly selected deletion mutants from the RV collection compared with WD repeat domain of the original *het-R *allele (A and B) of from the CE collection with the original *het-E *allele (C and D) were compared. Click here for file

Additional file 6 Evidences for occurrence of gene conversion. Click here for file

Additional file 7 Evidences for occurrence of unequal crossing overs. Click here for file

Additional file 8 Combinations of amino acids found at position 7, 9 25 and 27 of the WD40 repeat units in the *hnwd *gene family analysed here. Click here for file

Additional file 9 Nucleic and peptidic sequences of *het-R *mutant alleles affected in the HET or NACHT domain. Click here for file

Additional file 10 List of the accession numbers for the sequences used in this study. Click here for file
