# Supplementary material for: WD-repeat instability and diversification of the Podospora anserina hnwd non-self recognition gene family
Source: BMC Evol Biol. 2010 May 6;10:134. doi: 10.1186/1471-2148-10-134 (PMC2873952; doi:10.1186/1471-2148-10-134)
Supplement: Additional file 1 — Details of the primers used. [file 1471-2148-10-134-S1.PDF]

Additional file 1: Details of the primers used

Locus	Primer	Sequence	Orientation	Localisation*	Use
het-r	A	GCACCGGTTGGCAGTCTGG	Forward	+2388 to +2406	WD domain amplification
het-r	B	CCAGGCCCTTCTCGTGTTAGG	Reverse	+3170 to+3150	WD domain amplification
het-r	C	CCTGGAGCACCTCCTCC	Forward	-771 to -755	*het-r* locus cloning
het-r	D	CATGGGAGATGCTAGAATTCC	Reverse	+3219 to +3199	*het-r* locus cloning
het-r	E	TACGAACTCGACAGCTACTT	Forward	-190 to -171	HET domain sequencing
het-r	G	GAATGCATGCCTTCAGACG	Forward	+2474 to +2492	WD domain sequencing
het-r	H	CTCGAAGGCTATAGGAGTTC	Forward	+2871 to +2890	WD domain sequencing
het-r	I	CTGATCCAGCTAGGTTCTTCC	Reverse	+2446 to +2426	NACHT domain sequencing
het-r	K	GATAGAGGTCTTCCCTGAAG	Reverse	+553 to +534	HET domain sequencing
het-r	L	AGCTCTTCAGGGAAGACCTC	Forward	+530 to +549	NACHT domain sequencing
het-r	M	CTCGCTATTCGGTATCTTTGACG	Forward	+626 to +648	NACHT domain sequencing
het-r	N	GATCCAGACTTGAAGGAAAC	Forward	+1170 to +1189	NACHT domain and intron sequencing
het-r	O	GTTTGGGTAGGTCGATAACG	Reverse	+1239 to +1220	NACHT and HET domain sequencing
het-r	P	GATCTTGTCTTCTCGGATGG	Reverse	+1820 to +1801	NACHT domain sequencing
het-r	Q	CATCAGTCAGCCAAGGACTA	Forward	+1830 to +1849	Intron sequencing
het-d	het-D-F	GCTTCAGCACTTGTGTTTAGC	Forward	*het-D* : +2355 to +2375	WD domain cloning and sequencing
het-d	het-D-R	CGTTAGATCCAGATGCAATCAGC	Reverse	*het-D* : +3183 to +3161	WD domain cloning and sequencing
het-e	het-E-F	GGCTGGTCTAACAGTAGTATAGG	Forward	*het-E* : +2310 to +2332	WD domain cloning and sequencing
het-e	het-E-R	GTGTACAGGTTCCCGATGCC	Reverse	*het-E* : +2803 to+2784	WD domain cloning and sequencing
HNWD1	HNWD1-F	GAACGGAAGTGGATTGCTTTGG	Forward	*HNWD1* : +2453 to +2474	WD domain cloning and sequencing
HNWD1	HNWD1-R	CCAGACCCCTCCTGTGTCACG	Reverse	*HNWD1* : +4737 to +4717	WD domain cloning and sequencing
HNWD3	HNWD3-F	GTACGGGATCAGTAGAAATGC	Forward	*HNWD3* : +2529 to +2550	WD domain cloning and sequencing
HNWD3	HNWD3-R	CACCCCTTTGGTGGTAAACG	Reverse	*HNWD3* : +4198 to +4179	WD domain cloning and sequencing
NWD1	NWD1-F	CCTCATTAGAGAACTCTTCAAGAAGG	Forward	*NWD1* : +1789 to +1814	WD domain cloning and sequencing
NWD1	NWD1-R	CTACTGAGAACTAAAAAGGGCTGC	Reverse	*NWD1* : +3264 to +3241	WD domain cloning and sequencing
NWD2	NWD2-F	GTACAAGATAGTTGGAATGCGTGC	Forward	*NWD2* : +2768 to +2791	WD domain cloning and sequencing
NWD2	NWD2-R	CCCCTCCTTTGGCACGCTACG	Reverse	*NWD2* : +3479 to +3459	WD domain cloning and sequencing
NWDp1	NWDp1-F	CCTTATATTTATACCAAGTAGCAGC	Forward	*NWDp1* : -115 to -88	WD domain cloning and sequencing
NWDp1	NWDp1-R	GTATAGTCGAACGATAGATAGG	Reverse	*NWDp1* : +1069 to +1042	WD domain cloning and sequencing
NWDp2	NWDp2-F	TTCGCGATATATACCCGTTT	Forward	*NWDp2* : -191 to -172	WD domain cloning and sequencing
NWDp2	NWDp2-R	GCCCTTCCACCGTAAAAGTA	Reverse	*NWDp2* : +994 to +975	WD domain cloning and sequencing
NWDp3	NWDp3-F	GCTAGTTCGAACAACGGAAC	Forward	*NWDp3* : -255 to -236	WD domain cloning and sequencing
NWDp3	NWDp3-R	AATATACCCGGCATTGGTAA	Reverse	*NWDp3* : +965 to +946	WD domain cloning and sequencing

* : Primers are localized according to their position relative to the ATG codon of the corresponding locus in the sequenced strain (Espagne et al. 2008) as defined in Paoletti et al (2007), or according to the first nucleotide of the WD repeat domain for the pseudogene loci.
