# Supplementary material for: WD-repeat instability and diversification of the Podospora anserina hnwd non-self recognition gene family
Source: BMC Evol Biol. 2010 May 6;10:134. doi: 10.1186/1471-2148-10-134 (PMC2873952; doi:10.1186/1471-2148-10-134)

Additional file 2: Mapping of the deletions occurring in the dWD class of mutants of the RV collection.

A/ The WD repeat domain of the WT *het-R* allele includes six *Bgl*II sites in WD40 repeats number 1, 3, 4, 5, 6 and 10. *Bgl*II restriction of a PCR fragment amplified with primers 1 and 2 (arrowheads) results in a characteristic restriction pattern comprising DNA bands of 126 bp, 194 bp, 252bp, 463 bp and 504 bp.

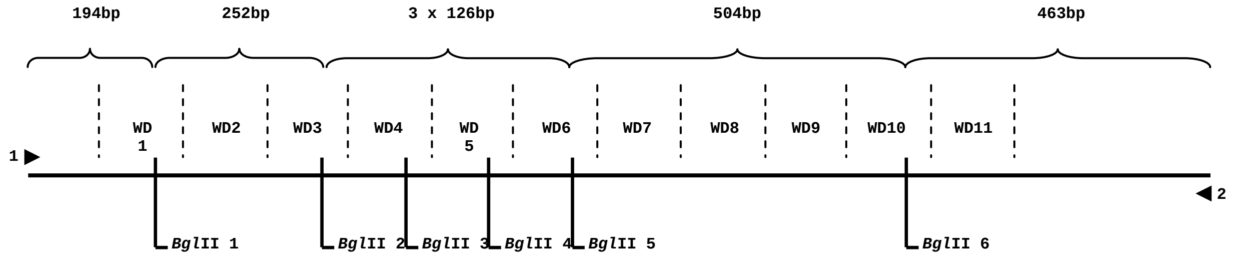

B/ Predicted *Bgl*II restriction profiles for the *het-R* deletion mutants :

For each subclass of deletion mutants (defined by the number of WD40 repeat unit deleted), and assuming deletions of a single DNA fragment including contiguous WD40 repeat units, we predicted all possible *Bgl*II restriction profiles of the WD repeat domain from the deletion mutants, amplified with primers A and B.. Each profile is named with a number corresponding to the subclass (number of deleted repeats, 1 to 11) of the deletion mutant and a letter, and is compared to the WT *het-R* allele at the top. (+) presence of the DNA band, (-) absence of the DNA band. The size of the bands is given in bp.

	*Bgl*II fragments observed in the *het-R* allele					*Bgl*II fragments generated by the deletion										
Profile name	504	463	252	194	126	841	715	630	589	572	531	446	405	378	337	320
het-R	+	+	+	+	+	-	-	-	-	-	-	-	-	-	-	-
1a	+	+	-	-	+	-	-	-	-	-	-	-	-	-	-	+
1b	+	+	-	+	+	-	-	-	-	-	-	-	-	-	-	-
1c	+	+	+	+	+	-	-	-	-	-	-	-	-	-	-	-
1d	-	+	+	+	+	-	-	-	-	-	-	-	-	+	-	-
1e	-	-	+	+	+	+	-	-	-	-	-	-	-	-	-	-
1f	+	-	+	+	+	-	-	-	-	-	-	-	-	-	+	-
2a	+	+	-	+	+	-	-	-	-	-	-	-	-	-	-	-
2b	+	+	+	+	+	-	-	-	-	-	-	-	-	-	-	-
2c	-	+	+	+	+	-	-	-	-	-	-	-	-	+	-	-
2d	-	+	+	+	+	-	-	-	-	-	-	-	-	-	-	-
2e	-	-	+	+	+	-	+	-	-	-	-	-	-	-	-	-
3a	+	+	-	+	+	-	-	-	-	-	-	-	-	-	-	-
3b	+	+	+	+	-	-	-	-	-	-	-	-	-	-	-	-
3c	-	+	+	+	+	-	-	-	-	-	-	-	-	+	-	-
3d	-	+	+	+	+	-	-	-	-	-	-	-	-	-	-	-
3[e]	-	-	+	+	+	-	-	-	+	-	-	-	-	-	-	-
4a	+	+	-	+	+	-	-	-	-	-	-	-	-	-	-	-
4b	-	+	-	+	-	-	-	+	-	-	-	-	-	-	-	-
4c	-	+	+	+	-	-	-	-	-	-	-	-	-	+	-	-
4d	-	+	+	+	+	-	-	-	-	-	-	-	-	-	-	-
5a	+	+	-	+	-	-	-	-	-	-	-	-	-	-	-	-
5b	-	+	+	+	-	-	-	-	-	-	-	-	-	-	-	-
5c	-	+	+	+	+	-	-	-	-	-	-	-	-	-	-	-
5d	-	-	+	+	+	-	-	-	-	-	-	-	-	-	+	-
6a	-	+	-	-	-	-	-	-	-	+	-	-	-	-	-	-
6b	-	+	-	+	-	-	-	-	-	-	-	-	-	+	-	-
6c	-	+	+	+	+	-	-	-	-	-	-	-	-	-	-	-
6d	-	-	+	+	+	-	-	-	-	-	-	-	-	-	+	-
7a	-	+	-	-	-	-	-	-	-	-	-	+		-	-	-
7b	-	+	+	+	-	-	-	-	-	-	-	-	-	-	-	-
7c	-	-	+	+	+	-	-	-	-	-	-	-	-	-	+	-
8a	-	+	-	-	-	-	-	-	-	-	-	-	-	-	-	+
8b	-	+	-	+	+	-	-	-	-	-	-	-	-	-	-	-
8c	-	-	-	+	-	-	-	-	+	-	-	-	-	-	-	-
8d	-	-	+	+	-	-	-	-	-	-	-	-	-	-	+	-
9a	-	+	-	+	-	-	-	-	-	-	-	-	-	-	-	-
10a	-	-	-	-	-	-	-	-	-	-	+	-	-	-	-	-
10b	-	-	-	+	-	-	-	-	-	-	-	-	-	-	+	-
11a	-	-	-	-	-	-	-	-	-	-	-	-	+	-	-	-

C/ Example of an agarose gel electrophoresis (1.2%) after *Bgl*II restriction WD repeat domains amplified with primers 1 and 2 from WT and different deletion mutants.

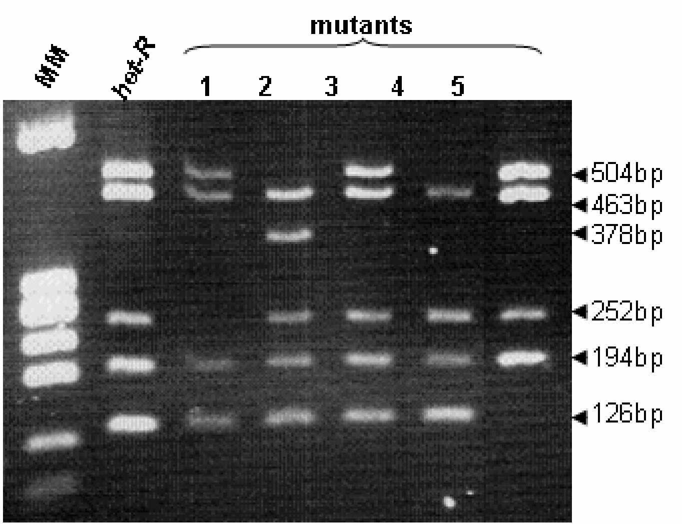

D/ Delineation of the intervals in which a single deletion can produce a given *Bgl*II restriction profile.
Because of the repeated nature of the WD repeat domain, a given restriction profile can be generated by different deletions. Consequently, for each *Bgl*II restriction profile described above, we defined intervals in which deletions could be initiated to produce the considered *Bgl*II restriction profile. All the intervals depicted above are named after the restriction profile they will produce when a deletion is initiated in them. The sizes of the DNA bands expected are given in bp.

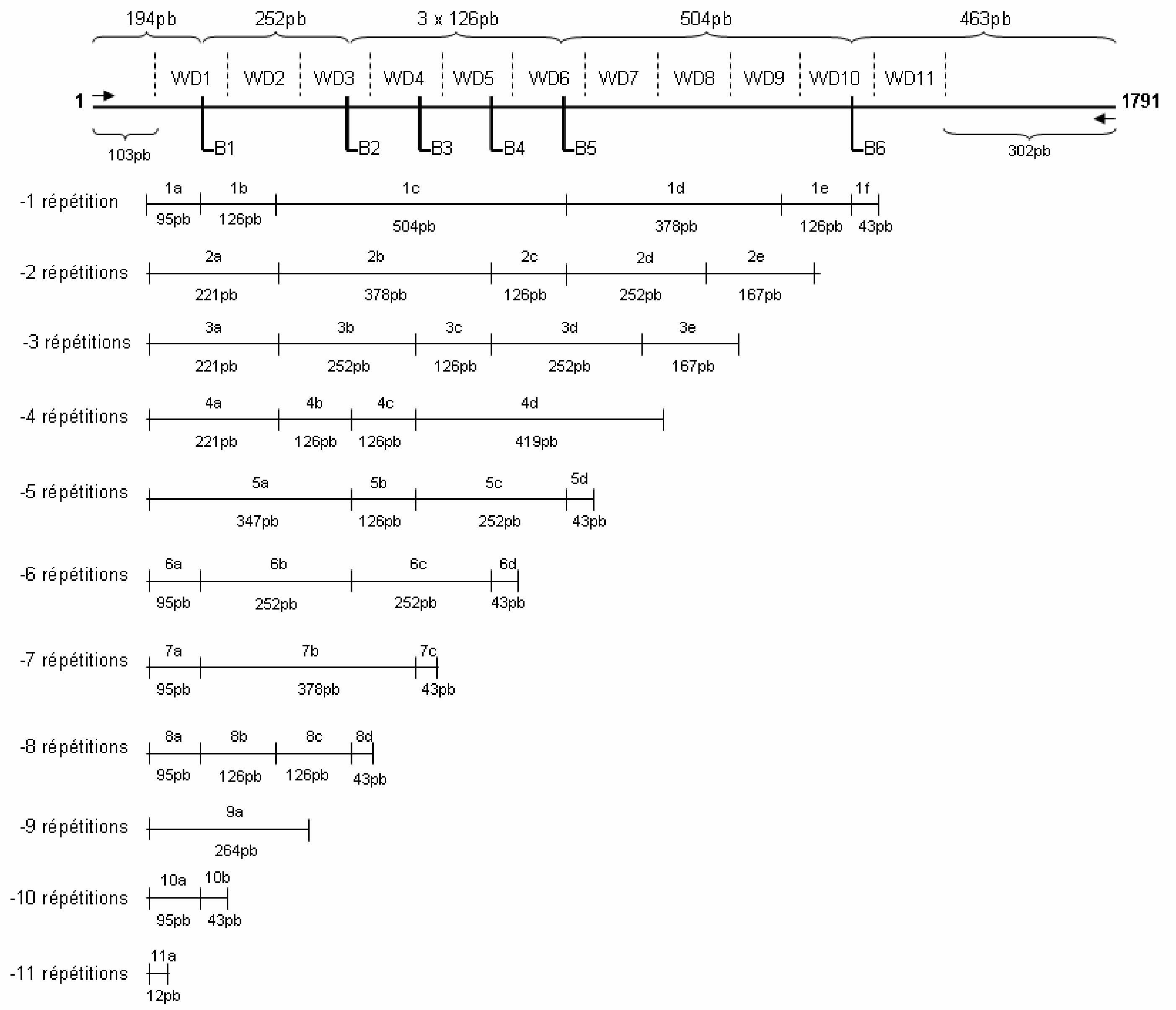

E/ Restriction profiles observed in the *het-R* deletion mutants. Only 7 mutants (highlighted) resulted in unpredicted BglII restriction profile

**Mutants of subclass -1**	Present in *het-R* allele					Generated by the deletion						
	504bp	463bp	252bp	194bp	126bp	967bp	841bp	715bp	589bp	531bp	378bp	337bp
RV-C2	-	+	+	+	+	-	-	-	-	-	+	-
RV-D1	-	-	+	+	+	+	-	-	-	-	-	-
RV-E2	+	-	+	+	+	-	-	-	-	-	-	+
RV-E4	+	+	+	+	+	-	-	-	-	-	-	-
RV-F4	+	+	-	+	+	-	-	-	-	-	-	-
RV-G3	+	+	+	+	+	-	-	-	-	-	-	-
RV-G5	-	+	+	+	+	-	-	-	-	-	+	-
RV-G8	+	+	+	+	+	-	-	-	-	-	-	-
RV-H2	-	-	+	+	+	+	-	-	-	-	-	-
RV-I1	+	+	-	+	+	-	-	-	-	-	-	-
RV-J2	+	+	+	+	+	-	-	-	-	-	-	-
RV-K4	+	+	+	+	+	-	-	-	-	-	-	-
RV-L2	+	+	+	+	+	-	-	-	-	-	-	-
RV-L7	+	+	+	+	+	-	-	-	-	-	-	-
RV-L14	+	-	+	+	+	-	-	-	-	-	-	+
RV-L15	+	+	+	+	+	-	-	-	-	-	-	-
RV-M9	+	+	+	+	+	-	-	-	-	-	-	-
RV-M15	-	-	+	+	+	-	+	-	-	-	-	-
RV-N5	-	+	+	+	+	-	-	-	-	-	+	-
RV-N8	+	+	+	+	+	-	-	-	-	-	-	-

**Mutants of subclass -2**	Present in *het-R* allele					Generated by the deletion						
	504bp	463bp	252bp	194bp	126bp	967bp	841bp	715bp	589bp	531bp	378bp	337bp
RV-A2	+	+	-	+	+	-	-	-	-	-	-	-
RV-A4	+	+	-	+	+	-	-	-	-	-	-	-
RV-F3	-	+	+	+	+	-	-	-	-	-	-	-
RV-G18	-	-	+	+	+	-	+	-	-	-	-	-
RV-H5	+	+	+	+	+	-	-	-	-	-	-	-
RV-H7	-	+	+	+	+	-	-	-	-	-	-	-
RV-I6	+	+	+	+	+	-	-	-	-	-	-	-
RV-I7	+	+	-	+	+	-	-	-	-	-	-	-
RV-K1	+	+	+	+	+	-	-	-	-	-	-	-
RV-K3	+	+	-	+	+	-	-	-	-	-	-	-
RV-K5	-	-	+	+	+	-	-	+	-	-	-	-
RV-K8	+	+	-	+	+	-	-	-	-	-	-	-
RV-L5	-	-	+	+	+	-	-	+	-	-	-	-
RV-L16	-	+	+	+	+	-	-	-	-	-	-	-
RV-M2	+	+	-	+	+	-	-	-	-	-	-	-
RV-M3	+	+	-	+	+	-	-	-	-	-	-	-
RV-M10	+	+	+	+	+	-	-	-	-	-	-	-
RV-N1	-	-	+	+	+	-	-	+	-	-	-	-

**Mutants of subclass -3**	Present in *het-R* allele					Generated by the deletion						
	504bp	463bp	252bp	194bp	126bp	967bp	841bp	715bp	589bp	531bp	378bp	337bp
RV-B1	+	+	-	+	+	-	-	-	-	-	-	-
RV-B5	-	-	+	+	+	-	-	-	+	-	-	-
RV-C3	-	-	+	+	+	-	-	-	+	-	-	-
RV-C5	-	+	+	+	+	-	-	-	-	-	+	-
RV-D2	-	+	+	+	+	-	-	-	-	-	-	-
RV-F5	+	+	+	+	-	-	-	-	-	-	-	-
RV-G4	-	-	+	+	+	-	-	+	-	-	-	-
RV-G9	-	+	+	+	+	-	-	-	-	-	-	-
RV-G11	+	+	-	+	+	-	-	-	-	-	-	-
RV-L1	+	+	-	+	+	-	-	-	-	-	-	-
RV-L3	+	+	-	+	+	-	-	-	-	-	-	-
RV-L4	+	+	-	+	+	-	-	-	-	-	-	-
RV-M4	-	-	+	+	+	-	-	-	+	-	-	-
RV-M5	-	-	+	+	+	-	-	-	+	-	-	-
RV-M14	+	+	+	+	-	-	-	-	-	-	-	-
RV-N6	-	+	+	+	+	-	-	-	-	-	+	-
RV-N7	+	+	+	+	-	-	-	-	-	-	-	-

**Mutants of subclass -4**	Present in *het-R* allele					Generated by the deletion						
	504bp	463bp	252bp	194bp	126bp	967bp	841bp	715bp	589bp	531bp	378bp	337bp
RV-B3	+	+	-	+	+	-	-	-	-	-	-	-
RV-C4	-	+	+	+	+	-	-	-	-	-	-	-
RV-F1	+	+	-	+	+	-	-	-	-	-	-	-
RV-G6	-	+	+	+	+	-	-	-	-	-	-	-
RV-G7	+	+	-	+	+	-	-	-	-	-	-	-
RV-G10	-	+	+	+	+	-	-	-	-	-	-	-
RV-H1	-	+	+	+	+	-	-	-	-	-	-	-
RV-H4	+	+	-	+	+	-	-	-	-	-	-	-
RV-H9	-	+	+	+	+	-	-	-	-	-	-	-
RV-I2	+	+	-	+	+	-	-	-	-	-	-	-
RV-I3	+	+	-	+	+	-	-	-	-	-	-	-
RV-L8	+	+	-	+	+	-	-	-	-	-	-	-
RV-M11	-	+	+	+	+	-	-	-	-	-	-	-

**Mutants of subclass -5**	Present in *het-R* allele					Generated by the deletion						
	504bp	463bp	252bp	194bp	126bp	967bp	841bp	715bp	589bp	531bp	378bp	337bp
RV-C7	+	+	-	+	-	-	-	-	-	-	-	-
RV-F2	-	+	+	+	-	-	-	-	-	-	-	-
RV-G15	-	+	+	+	+	-	-	-	-	-	-	-
RV-L6	-	+	+	+	-	-	-	-	-	-	-	-
RV-L13	+	+	-	+	-	-	-	-	-	-	-	-
RV-M13	+	+	-	+	-	-	-	-	-	-	-	-
RV-N3	-	-	+	+	+	-	-	-	-	-	-	+

Mutants of subclass -6	Present in *het-R* allele					Generated by the deletion						
	504bp	463bp	252bp	194bp	126bp	967bp	841bp	715bp	589bp	531bp	378bp	337bp
RV-A3	-	+	-	+	-	-	-	-	-	-	+	-
RV-E7	-	+	+	+	+	-	-	-	-	-	-	-
RV-F6	-	-	+	+	+	-	-	-	-	-	-	+
RV-G14	-	+	+	+	+	-	-	-	-	-	-	-
RV-J3	-	+	-	+	-	-	-	-	-	-	+	-
RV-K7	-	+	-	+	-	-	-	-	-	-	+	-
RV-M16	-	+	-	+	-	-	-	-	-	-	+	-
RV-M17	-	-	+	+	+	-	-	-	-	-	-	+

Mutants of subclass -7	Present in *het-R* allele					Generated by the deletion						
	504bp	463bp	252bp	194bp	126bp	967bp	841bp	715bp	589bp	531bp	378bp	337bp
RV-B2	-	+	+	+	-	-	-	-	-	-	-	-
RV-G13	-	+	+	+	-	-	-	-	-	-	-	-
RV-J1	-	+	-	+	+	-	-	-	-	-	-	-
RV-K2	-	+	+	+	-	-	-	-	-	-	-	-
RV-K6	-	-	+	+	+	-	-	-	-	-	-	+

Mutants of subclass -8	Present in *het-R* allele					Generated by the deletion						
	504bp	463bp	252bp	194bp	126bp	967bp	841bp	715bp	589bp	531bp	378bp	337bp
RV-E3	-	-	-	+	-	-	-	+	-	-	-	-
RV-H8	-	-	-	+	-	-	-	+	-	-	-	-

Mutants of subclass -9	Present in *het-R* allele					Generated by the deletion						
	504bp	463bp	252bp	194bp	126bp	967bp	841bp	715bp	589bp	531bp	378bp	337bp
RV-G1	-	+	-	+	-	-	-	-	-	-	-	-
RV-G16	-	+	-	+	-	-	-	-	-	-	-	-
RV-M6	-	+	-	+	-	-	-	-	-	-	-	-
RV-N4	-	+	-	+	-	-	-	-	-	-	-	-

Mutants in subclass -10	Present in *het-R* allele					Generated by the deletion						
	504bp	463bp	252bp	194bp	126bp	967bp	841bp	715bp	589bp	531bp	378bp	337bp
RV-H6	-	-	-	-	-	-	-	-	-	+	-	-

F/ Deletions occur all along the WD repeat domain with a frequency compatible with random initiation of deletions.
Schematic representation of the PCR amplicon comprising the WD repeat domain of the active *het-R* allele. The primers are represented by arrowheads. The WD40 repeat units and the *Bgl*II restriction sites are localized along the sequence. The sizes of the fragments generated by *Bgl*II restriction are indicated at the top. Bottom: For the three subclasses of mutants comprising the most individuals, the intervals in which initiation of deletions occurs to generate a given *Bgl*II restriction profile are indicated by horizontal bars and their lengths indicated in bp. On top are indicated the observed number of mutants occurring in each interval and in brackets the corresponding number E expected if deletions occur at random. E depends on the length of the interval and is : E=(N x l)/L where N is the total number of mutants in a sub class, l the length of the interval and L the length of the WD domain.

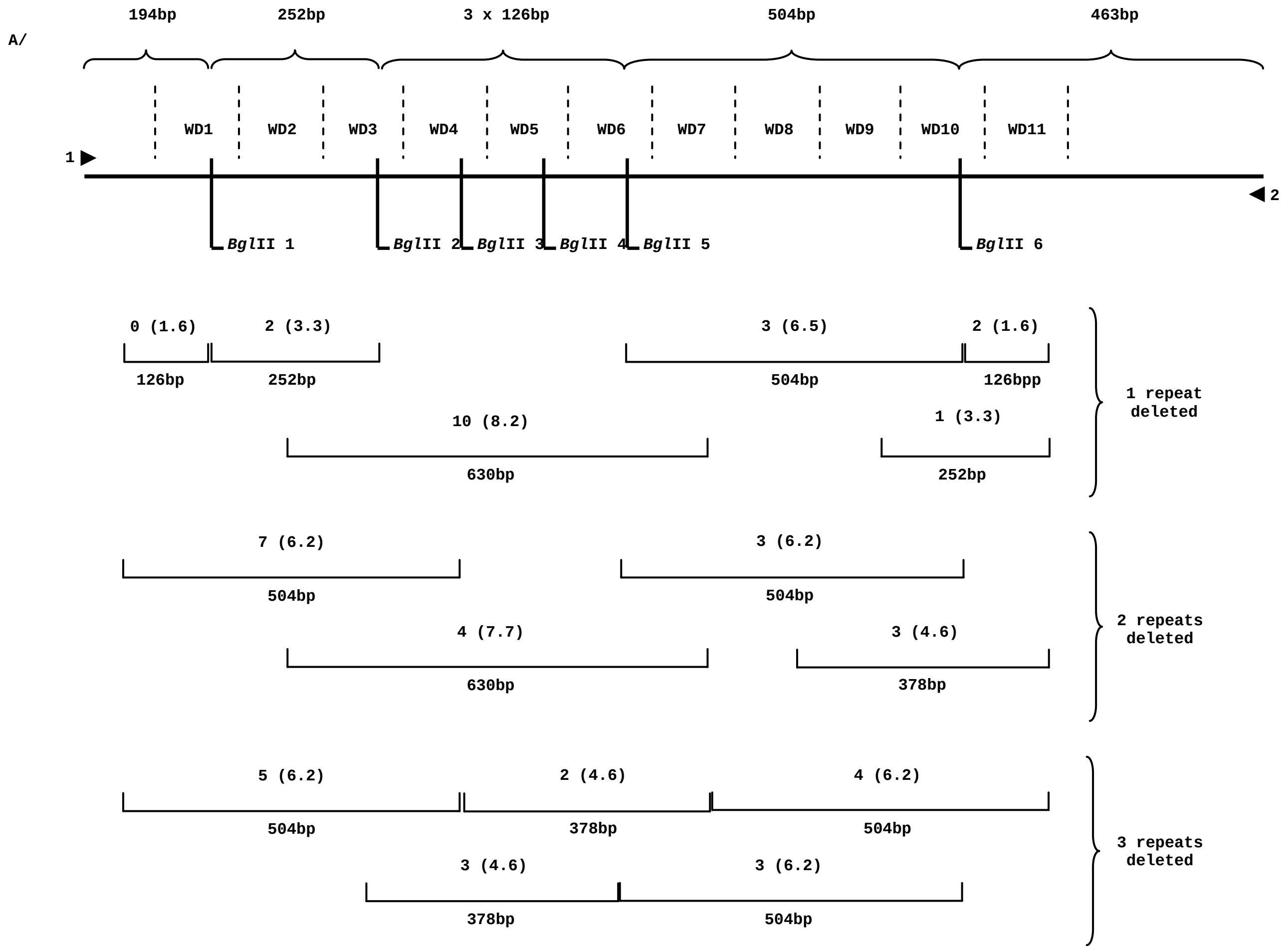

Supplement: Additional file 2 — Mapping of the deletions occurring in the dWD class of mutants of the RV collection. [file 1471-2148-10-134-S2.PDF]
