# Supplementary material for: WD-repeat instability and diversification of the Podospora anserina hnwd non-self recognition gene family
Source: BMC Evol Biol. 2010 May 6;10:134. doi: 10.1186/1471-2148-10-134 (PMC2873952; doi:10.1186/1471-2148-10-134)
Supplement: Additional file 3 — Schematic representation of the parental WD repeat domains of the hnwd gene family members. [file 1471-2148-10-134-S3.PDF]

Additional file 3: Schematic representation of the parental WD repeat domains of the *HNWD* gene family members

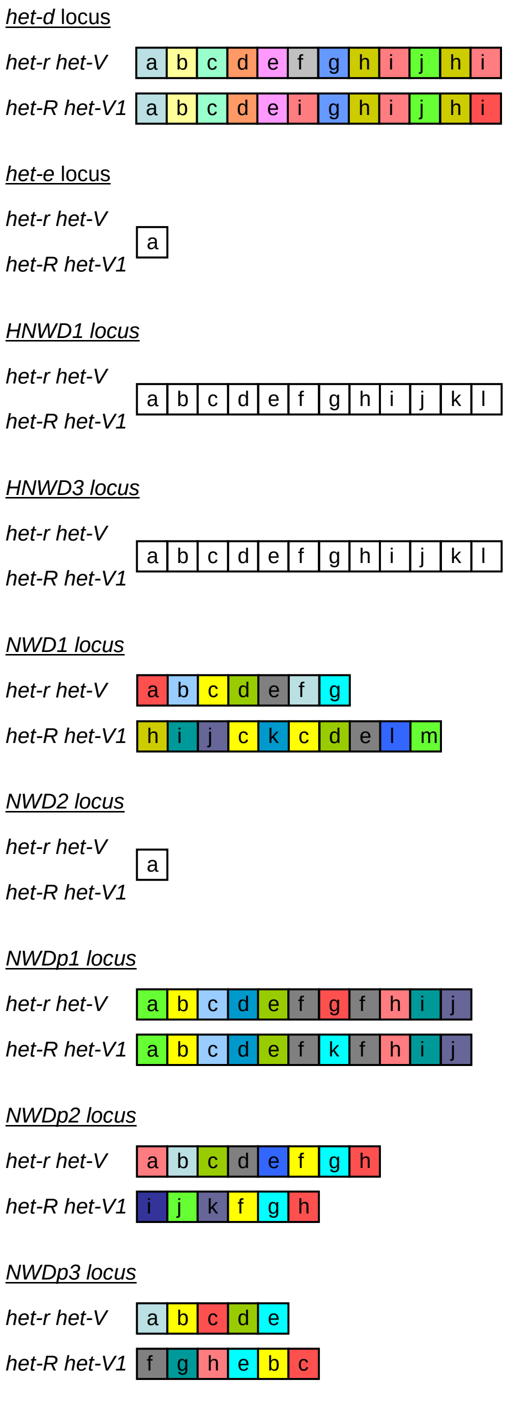

Supplemental fig. S3:

WD repeat domain organisation of the members of the *NWD* gene family in the *het-R het-V1* and *het-r het-V* strains. At each locus where alternative alleles are found, repeat units with the same nucleic sequence are represented with the same colour and letter.
