# Supplementary material for: WD-repeat instability and diversification of the Podospora anserina hnwd non-self recognition gene family
Source: BMC Evol Biol. 2010 May 6;10:134. doi: 10.1186/1471-2148-10-134 (PMC2873952; doi:10.1186/1471-2148-10-134)

Additional file 4:
Neighbor joining phylogenetic tree constructed with the nucleic sequences corresponding to WD40 repeat units from all members of the *P. anserina NWD* gene family, along with the new complex WD40 repeat unit sequences from the RV and CE collections (black boxes on figure 3). WD40 repeat units are named after their gene of origin and their position in the repeat array. The tree was constructed based on the number of differences with the program MEGA3. Only Internal branch test values over 90, Calculated over 1000 replicates, are presented.
Mutant repeat units are labeled with the name of their mutant of origin and their position in the mutant repeat array.

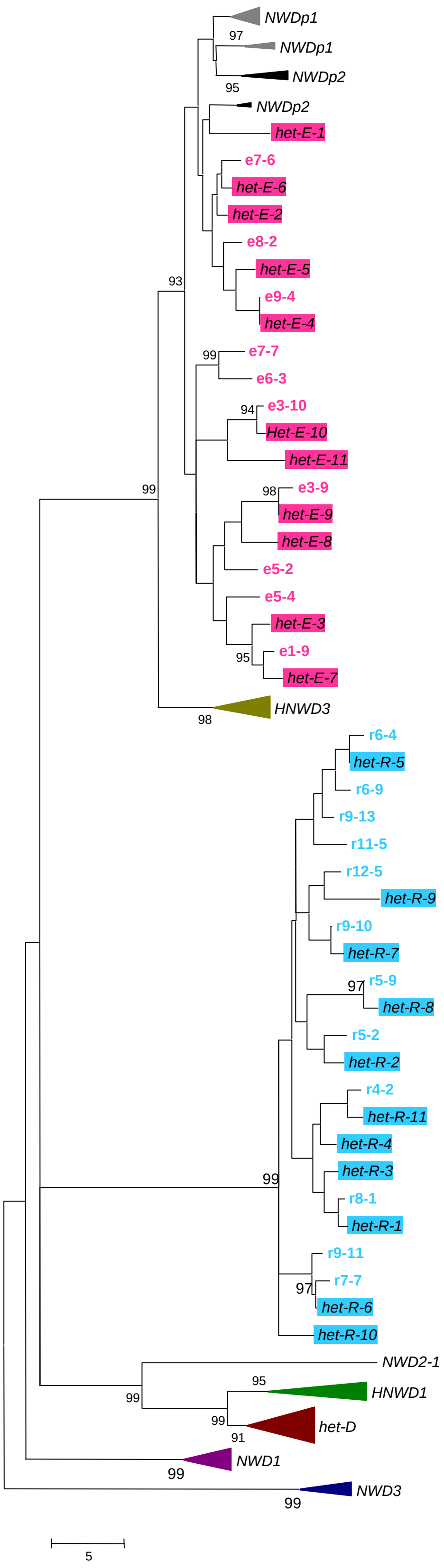

Supplement: Additional file 4 — Neighbor joining phylogenetic tree constructed with the nucleic sequences corresponding to WD40 repeat units from all members of the P. anserina nwd gene family, along with the new complex WD40 repeat unit sequences from the RV and CE collections. [file 1471-2148-10-134-S4.PDF]
