# Supplementary material for: WD-repeat instability and diversification of the Podospora anserina hnwd non-self recognition gene family
Source: BMC Evol Biol. 2010 May 6;10:134. doi: 10.1186/1471-2148-10-134 (PMC2873952; doi:10.1186/1471-2148-10-134)
Supplement: Additional file 5 — Protein sequences of WD repeat domains of randomly selected deletion mutants from the RV collection compared with WD repeat domain of the original het-R allele (A and B) of from the CE collection with the original het-E allele (C and D) were compared. [file 1471-2148-10-134-S5.PDF]

Additional file 5:

Protein sequences of WD repeat domains of randomly selected deletion mutants from the RV collection compared with WD repeat domain of the original *het-R* allele (A and B) of from the CE collection with the original *het-E* allele (C and D) were compared. For each sequence alignment the start of each repeat is highlighted in yellow and the new chimeric WD40 repeat unit is written in red. E/ Schematic representation of the architecture of these repeat domains.

A/

```
r10                1 LEGHNGSVYSVAFSADGQRLASGAGDRTVKIWDPASGQCFQTLEGHNGSV     50
                     ||||||||||||||||||||||||||||||||||||||||||||||||||
het-R              1 LEGHNGSVYSVAFSADGQRLASGAGDRTVKIWDPASGQCFQTLEGHNGSV     50

r10               51 YSVAFSPDGQRLASGAVDDTVKIWDPASGQCLQTLEGHNGSVYSVAFSAD    100
                     ||||||||||||||||||||||||||||||||||||||||||||||||||
het-R             51 YSVAFSPDGQRLASGAVDDTVKIWDPASGQCLQTLEGHNGSVYSVAFSAD    100

r10              101 GQRLASGAGDDTVKIWDPASGQCLQTLEGHRGSVSSVAFSADGQRLASGA    150
                     ||||||||||||||||||||||||||||||||||||||||||||||||||
het-R            101 GQRLASGAGDDTVKIWDPASGQCLQTLEGHRGSVSSVAFSADGQRLASGA    150

r10              151 VDRTVKIWDPASGQCLQTLEGHTGSVSSVAFSP-----------------    183
                     |||||||||||||||||||||||||||||||||
het-R            151 VDRTVKIWDPASGQCLQTLEGHTGSVSSVAFSPDGQRFASGVVDDTVKIW    200

r10              184 --------------------------------------------------    183

het-R            201 DPASGQCLQTLEGHRGSVSSVAFSPDGQRFASGAGDRTIKIWDPASGQCL    250

r10              184 -----------------DGQRFASGAGDDTVKIWDPASGQCLQTLESHNG    216
                                      |||||||||||||||||||||||||||||||||
het-R            251 QTLEGHRGWVYSVAFSADGQRFASGAGDDTVKIWDPASGQCLQTLESHNG    300

r10              217 SVSSVAFSPDGQRLASGADDDTVKIWDPASGQCLQTLEGHKGLVYSVTFS    266
                     ||||||||||||||||||||||||||||||||||||||||||||||||||
het-R            301 SVSSVAFSPDGQRLASGADDDTVKIWDPASGQCLQTLEGHKGLVYSVTFS    350

r10              267 ADGQRLASGAGDDTVKIWDPASGQCLQTLEGHRGSVHSVAFSPDGQRFAS    316
                     ||||||||||||||||||||||||||||||||||||||||||||||||||
het-R            351 ADGQRLASGAGDDTVKIWDPASGQCLQTLEGHRGSVHSVAFSPDGQRFAS    400

r10              317 GAVDDTVKIWDPASGQCLQTLEGHNGSVSSVAFSADGQRLASGAVDCTVK    366
                     ||||||||||||||||||||||||||||||||||||||||||||||||||
het-R            401 GAVDDTVKIWDPASGQCLQTLEGHNGSVSSVAFSADGQRLASGAVDCTVK    450

r10              367 IWDPASGQCLQT    378
                     ||||||||||||
het-R            451 IWDPASGQCLQT    462
```

Chimeric WD40 unit

>r10-5

LEGHTGSVSSVAFSPDGQRFASGAGDDTVKIWDPASGQCLQT

B/

```
R11              1 LEGHNGSVYSVAFSADGQRLASGAGDRTVKIWDPASGQCFQTLEGHNGSV    50
                   ||||||||||||||||||||||||||||||||||||||||||||||||||
het-R            1 LEGHNGSVYSVAFSADGQRLASGAGDRTVKIWDPASGQCFQTLEGHNGSV    50

r11             51 YSVAFSPDGQRLASGADDDTVKIWDPASGQCLQTLEGHRGSVSSVAFSAD   100
                   |||||||||||||||| ||||||||||||||||||||| ||| |||||||
het-R           51 YSVAFSPDGQRLASGAVDDTVKIWDPASGQCLQTLEGHNGSVYSVAFSAD   100

r11            101 GQRLASGAGDDTVKIWDPASGQCLQTLEGRRGSVSSVAFSADGQRLASGA   150
                   ||||||||||||||||||||||||||||| ||||||||||||||||||||
het-R          101 GQRLASGAGDDTVKIWDPASGQCLQTLEGHRGSVSSVAFSADGQRLASGA   150

r11            151 VDRTVKIWDPASGQCLQTLEGHRGSVSSVAFS                      183
                   |||||||||||||||||||||| |||||||||------------------
het-R          151 VDRTVKIWDPASGQCLQTLEGHTGSVSSVAFSPDGQRFASGVVDDTVKIW   200

r11            183                                                   183
                   --------------------------------------------------
het-R          201 DPASGQCLQTLEGHRGSVSSVAFSPDGQRFASGAGDRTIKIWDPASGQCL   250

r11            183                                                   183
                   --------------------------------------------------
het-R          251 QTLEGHRGWVYSVAFSADGQRFASGAGDDTVKIWDPASGQCLQTLESHNG   300

r11            183                                                   183
                   --------------------------------------------------
het-R          301 SVSSVAFSPDGQRLASGADDDTVKIWDPASGQCLQTLEGHKGLVYSVTFS   350

r11            183 ADGQRLASGAGDDTVKIWDPASGQCLQTLEGHRGSVHSVAFSPDGQRFAS   232
                   ||||||||||||||||||||||||||||||||||||||||||||||||||
het-R          351 ADGQRLASGAGDDTVKIWDPASGQCLQTLEGHRGSVHSVAFSPDGQRFAS   400

r11            233 GAVDDTVKIWDPASGQCLQTLEGHNGSVSSVAFSADGQRLASGAVDCTVK   282
                   ||||||||||||||||||||||||||||||||||||||||||||||||||
het-R          401 GAVDDTVKIWDPASGQCLQTLEGHNGSVSSVAFSADGQRLASGAVDCTVK   450

r11            283 IWDPASGQCLQT   294
                   ||||||||||||
het-R          451 IWDPASGQCLQT   462
```

Chimeric WD40 repeat unit

>r11-5

LEGHRGSVSSVAFSADGQRLASGAGDDTVKIWDPASGQCLQT

C/

```
e9           1 LEGHGSSVLSVAFSPDGQRVASGSDDKTIKIWDTASGTGTQTLEGHGGSV   50
               ||||||||||||||||||||||||||||||||||||||||||||||||||
Het-E        1 LEGHGSSVLSVAFSPDGQRVASGSDDKTIKIWDTASGTGTQTLEGHGGSV   50

e9          51 WSVAFTPDGQRVASGSDDKTIKIWDAASGTCTQTLEGHGGRVQSVAFSPD  100
               |||||:||||||||||||||||||||||||||||||||||||||||||||
Het-E       51 WSVAFSPDGQRVASGSDDKTIKIWDAASGTCTQTLEGHGGRVQSVAFSPD  100

e9         101 GQRVASGSDDHTIKIRDAASGTCTQTLEGHGSSVLSVAFSPDGQRVASGS  150
               |||||||||||||||.||||||||||||||||||||||||||||||||||
Het-E      101 GQRVASGSDDHTIKIWDAASGTCTQTLEGHGSSVLSVAFSPDGQRVASGS  150

e9         151 GDKTIKIWDTA---------------------------------------  161
               |||||||||||
Het-E      151 GDKTIKIWDTASGTCTQTLEGHGNSVWSVAFSPDGQRVASGSGDKTIKIW  200

e9         162 --------------------------------------------------  161

Het-E      201 DTASGTCTQTLEGHGGSVWSVAFSPDGQRVASGSDDKTIKIWDTASGTCT  250

e9         162 -------------------------------------SGTCTQTLEGHGD  174
                                                    |||||||||||||
Het-E      251 QTLEGHGGWVQSVVFSPDGQRVASGSDDHTIKIWDAVSGTCTQTLEGHGD  300

e9         175 SVWSVAFSPDGQRVASGSIDDTIKIWDAASGTCTQTLEGHGGWVHSVAFS  224
               ||||||||||||||||||||.|||||||||||||||||||||||||||||
Het-E      301 SVWSVAFSPDGQRVASGSIDGTIKIWDAASGTCTQTLEGHGGWVHSVAFS  350

e9         225 PDGQRVASGSIDGTIKIWDAASGTCTQTLEGHGGWVQSVAFSPDGQRVAS  274
               ||||||||||||||||||||||||||||||||||||||||||||||||||
Het-E      351 PDGQRVASGSIDGTIKIWDAASGTCTQTLEGHGGWVQSVAFSPDGQRVAS  400

e9         275 GSSDKTIKIWDTASGTCTQTLEGHGGWVQSVAFSPDGQRVASGSSDNTIK  324
               ||||||||||||||||||||||||||||||||||||||||||||||||||
Het-E      401 GSSDKTIKIWDTASGTCTQTLEGHGGWVQSVAFSPDGQRVASGSSDNTIK  450

e9         325 IWDTASGTCTQT    336
               ||||||||||||
Het-E      451 IWDTASGTCTQT    462
```

Chimeric WD40 repeat unit
>e9-4
LEGHGSSVLSVAFSPDGQRVASGSGDKTIKIWDTASGTCTQT

D/

```
e8                  1 LEGHGSSVLSVAFSPDGQRVASGSDDKTIKIWDTASGTGTQTLEGHGGSV     50
                      ||||||||||||||||||||||||||||||||||||||||||||||||||
Het-E               1 LEGHGSSVLSVAFSPDGQRVASGSDDKTIKIWDTASGTGTQTLEGHGGSV     50

e8                 51 W-------------------------------------------------     51
                      |
Het-E              51 WSVAFSPDGQRVASGSDDKTIKIWDAASGTCTQTLEGHGGRVQSVAFSPD    100

e8                 52 -----------------------------------SVAFSPDGQRVASGS     66
                                                         |||||||||||||||
Het-E             101 GQRVASGSDDHTIKIWDAASGTCTQTLEGHGSSVLSVAFSPDGQRVASGS    150

e8                 67 GDKTIKIWDTASGTCTQTLEGHGNSVWSVAFSPDGQRVASGSGDKTIKIW    116
                      ||||||||||||||||||||||||||||||||||||||||||||||||||
Het-E             151 GDKTIKIWDTASGTCTQTLEGHGNSVWSVAFSPDGQRVASGSGDKTIKIW    200

e8                117 DTASGTCTQTLEGHGGSVWSVAFSPDGQRVASGSDDKTIKIWDTASGTCT    166
                      ||||||||||||||||||||||||||||||||||||||||||||||||||
Het-E             201 DTASGTCTQTLEGHGGSVWSVAFSPDGQRVASGSDDKTIKIWDTASGTCT    250

e8                167 QTLEGHGGWVQSVVFSPDGQRVASGSDDHTIKIWDAVSGTCTQTLEGHGD    216
                      ||||||||||||||||||||||||||||||||||||||||||||||||||
Het-E             251 QTLEGHGGWVQSVVFSPDGQRVASGSDDHTIKIWDAVSGTCTQTLEGHGD    300

e8                217 SVWSVAFSPDDQRVASGSIDGTIKIWDAASGTCTQTLEGHGGWVHSVAFS    266
                      ||||||||||.|||||||||||||||||||||||||||||||||||||||
Het-E             301 SVWSVAFSPDGQRVASGSIDGTIKIWDAASGTCTQTLEGHGGWVHSVAFS    350

e8                267 PDGQRVASGSIDGTIKIWDAASGTCTQTLEGHGGWVQSVAFSPDGQRVAS    316
                      ||||||||||||||||||||||||||||||||||||||||||||||||||
Het-E             351 PDGQRVASGSIDGTIKIWDAASGTCTQTLEGHGGWVQSVAFSPDGQRVAS    400

e8                317 GSSDKTIKIWDTASGTCTQTLEGHGGWVQSVAFSPDGQRVASGSSDNTIK    366
                      ||||||||||||||||||||||||||||||||||||||||||||||||||
Het-E             401 GSSDKTIKIWDTASGTCTQTLEGHGGWVQSVAFSPDGQRVASGSSDNTIK    450

e8                367 IWDTASGTCTQT    378
                      ||||||||||||
Het-E             451 IWDTASGTCTQT    462
```

Chimeric WD40 repeat unit

> e8-2

LEGHGGSVWSVAFSPDGQRVASGSGDKTIKIWDTASGTCTQT

E/

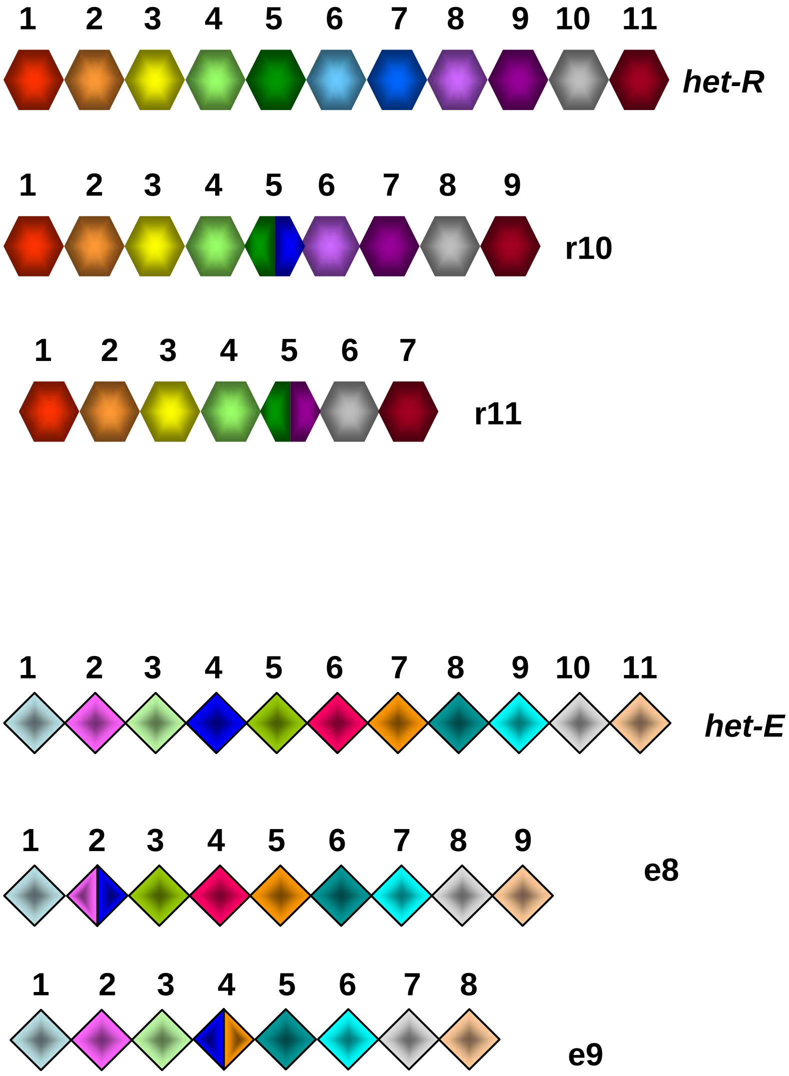
1 2 3 4 5 6 7 8 9 10 11
het-R
1 2 3 4 5 6 7 8 9
r10
1 2 3 4 5 6 7
r11
1 2 3 4 5 6 7 8 9 10 11
het-E
1 2 3 4 5 6 7 8 9
e8
1 2 3 4 5 6 7 8
e9
