# Supplementary material for: WD-repeat instability and diversification of the Podospora anserina hnwd non-self recognition gene family
Source: BMC Evol Biol. 2010 May 6;10:134. doi: 10.1186/1471-2148-10-134 (PMC2873952; doi:10.1186/1471-2148-10-134)
Supplement: Additional file 6 — Evidences for occurrence of gene conversion. [file 1471-2148-10-134-S6.PDF]

Additional file 6:

ClustalW alignment of the WD40 repeat units of the original *het-R* allele and the mutant WD40 repeat sequence r4-2 from mutant r4. Positions differing between the *het-R-2* and the mutant sequence are highlighted in green. At polymorphic sites between repeats, positions highlighted in grey are identical to the mutant sequence. The stretch of sequence in red is identical between the mutant sequence r4-2 and the het-R-4 repeat units and could be the result of gene conversion.

```
het-R-1         CTCGAAGGCCATAATGGCTCGGTTTACTCGGTCGCCTTTTCGGCGGATGGCCAGCGGCTC 60
het-R-3         CTCGAAGGCCATAATGGCTCGGTTTACTCGGTCGCCTTTTCGGCGGATGGCCAGCGGCTC 60
het-R-7         CTCGAAGGCCATAGGGGCTGGGTTTACTCGGTCGCCTTTTCGGCGGATGGCCAGCGGTTC 60
het-R-9         CTCGAAGGCCATAAGGGCTTGGTTTACTCGGTTACCTTTTCGGCGGATGGCCAGCGGCTC 60
het-R-6         CTCGAAGGCCATAGGGGCTCGGTTTCCTCGGTCGCCTTTTCGCCGGATGGCCAGCGGTTC 60
het-R-5         CTCGAAGGCCATACGGGCTCGGTTTCCTCGGTCGCCTTTTCGCCGGATGGCCAGCGGTTC 60
het-R-10        CTCGAAGGCCATAGGGGCTCGGTTCACTCGGTCGCCTTTTCGCCGGATGGCCAGCGGTTC 60
het-R-8         CTTGAAAGCCATAATGGCTCGGTTTCCTCGGTCGCCTTTTCGCCGGATGGCCAGCGGCTC 60
het-R-11        CTCGAAGGCCATAATGGCTCGGTTTCCTCGGTCGCCTTTTCGGCGGATGGCCAGCGGCTC 60
het-R-4         CTCGAAGGCCATAGGGGCTCGGTTTCCTCGGTCGCCTTTTCGGCGGATGGCCAGCGGCTC 60
het-R-2         CTCGAAGGCCATAATGGCTCGGTGTACTCGGTCGCCTTTTCGCCGGATGGCCAGCGGCTC 60
r4-2            CTCGAAGGCCATAATGGCTCGGTTTCCTCGGTCGCCTTTTCGGCGGATGGCCAGCGGCTC 60
                ** *** ******  **** ***   ******  ******** ************** **

het-R-1         GCATCCGGTGCAGGCGACCGTACCGTCAAGATCTGGGATCCCGCCTCGGGACAATGCTTC 120
het-R-3         GCATCCGGTGCAGGCGACGATACCGTCAAGATCTGGGATCCCGCCTCGGGACAATGCCTC 120
het-R-7         GCATCCGGTGCAGGCGACGATACCGTCAAAATCTGGGATCCCGCCTCGGGACAATGCCTC 120
het-R-9         GCATCTGGTGCAGGCGACGATACCGTCAAAATCTGGGATCCCGCCTCGGGACAATGCCTC 120
het-R-6         GCATCCGGTGCAGGCGACCGTACCATCAAGATCTGGGATCCCGCCTCGGGACAATGCCTC 120
het-R-5         GCATCTGGTGTAGTCGACGATACCGTCAAGATCTGGGATCCCGCCTCGGGACAATGCCTC 120
het-R-10        GCATCTGGTGCAGTCGACGATACCGTCAAGATCTGGGATCCCGCCTCGGGACAATGCCTC 120
het-R-8         GCATCTGGTGCAGACGACGATACCGTCAAAATCTGGGATCCCGCCTCGGGACAATGCCTC 120
het-R-11        GCATCTGGTGCAGTCGACTGTACCGTCAAAATCTGGGATCCCGCCTCGGGACAATGCCTC 120
het-R-4         GCATCCGGTGCAGTCGACCGTACCGTCAAGATCTGGGATCCCGCCTCGGGACAATGCCTC 120
het-R-2         GCATCTGGTGCAGTCGACGATACCGTCAAAATCTGGGATCCCGCCTCGGGACAATGCCTC 120
r4-2            GCATCCGGTGCAGTCGACCGTACCGTCAAAATCTGGGATCCCGCCTCGGGGCAATGCCTC 120
                ***** **** ** ****  **** **** ******************** ****** **

het-R-1         CAGACG 126
het-R-3         CAGACG 126
het-R-7         CAGACG 126
het-R-9         CAGACG 126
het-R-6         CAGACG 126
het-R-5         CAGACG 126
het-R-10        CAGACG 126
het-R-8         CAGACA 126
het-R-11        CAGACG 126
het-R-4         CAGACG 126
het-R-2         CAGACG 126
r4-2            CAGACG 126
                *****
```
