# Supplementary material for: WD-repeat instability and diversification of the Podospora anserina hnwd non-self recognition gene family
Source: BMC Evol Biol. 2010 May 6;10:134. doi: 10.1186/1471-2148-10-134 (PMC2873952; doi:10.1186/1471-2148-10-134)
Supplement: Additional file 7 — Evidences for occurrence of unequal crossing overs. [file 1471-2148-10-134-S7.PDF]

Additional file 7:

A/ ClustalW alignment of the WD40 repeat units of *het-E* and the mutant unit sequence e6-3 from mutant e6 (highlighted in light grey). At polymorphic positions, nucleotides identical to the mutant sequence are highlighted in yellow. The stretch of sequence written in red are identical to the mutant sequence, which could be a chimera between the 5' end of the third WD40 repeat and the 3' end of the second WD40 repeat units of the wild type allele. The break point could be at the A at position 49 highlighted in red. B/ Schematic representation of the possible unequal crossing over resulting in the formation of the e6 allele.

A/

```
het-E-4         TTAGAGGGCCATGGCGGCAGGGTCCAGTCGGTCGCGTTCTCGCCGGACGGCCAGCGCGTG 60
e6-3            TTAGAGGGCCATGGCGGCAGGGTCCAGTCGGTCGCGTTCTCGCCGGACGGCCAGCGCGTG 60
het-E-3         TTAGAGGGCCATGGCGGCAGGGTCCAGTCGGTCGCGTTCTCGCCGGACAGCCAGCGCGTG 60
het-E-2         TTAGAGGGCCATGGCGGCTCGGTCTGGTCGGTCGCGTTCTCGCCGGACGGCCAGCGCGTG 60
het-E-8         TTAGAGGGCCATGGCGGCTGGGTCCAGTCGGTCGTGTTCTCGCCGGACGGCCAGCGCGTG 60
het-E-5         TTAGAGGGCCATGGAAGCTCGGTCTTGTCGGTCGCGTTCTCGCCGGACGGCCAGCGCGTG 60
het-E-6         TTAGAGGGCCATGGCAACTCGGTCTGGTCGGTCGCGTTCTCGCCGGACGGCCAGCGCGTG 60
het-E-1         TTAGAGGGCCATGGCAGCTCGGTCTTGTCGGTCGCGTTCTCGCCGGACGGCCAGCGCGTG 60
het-E-7         TTAGAGGGCCATGGCGGCTCGGTCTGGTCGGTCGCGTTCTCGCCGGACGGCCAGCGCGTG 60
het-E-11        TTAGAGGGCCATGGCGGCTGGGTCCAGTCGGTCGCGTTCTCGCCGGACGGCCAGCGTGTG 60
het-E-12        TTAGAGGGCCATGGCGGCTGGGTCCAGTCGGTCGCGTTCTCGCCGGACGGCCAGCGTGTG 60
het-E-9         TTAGAGGGCCATGGCGACTCGGTCTGGTCGGTCGCGTTCTCGCCGGACGGCCAGCGCGTG 60
het-E-10        TTAGAGGGCCATGGCGGCTGGGTCCACTCGGTCGCGTTCTCGCCGGACGGCCAGCGCGTG 60
                **************   *  ****   ******* ************* ******* ***

het-E-4         GCATCGGGCTCGGACGACCACACCATTAAGATCTGGGATGCGGCATCGGGAACCTGTACA 120
e6-3            GCATCGGGCTCGGACGACCACACCATTAAGATCTGGGATGCGGCATCGGGAACCTGTACA 120
het-E-3         GCATCGGGCTCGGACGACAAGACCATCAAGATCTGGGATGCGGCATCGGGAACCTGTACA 120
het-E-2         GCATCGGGCTCGGACGACAAGACCATCAAGATCTGGGATGCGGCATCGGGAACCTGTACA 120
het-E-8         GCATCGGGCTCGGACGACCACACCATTAAGATCTGGGATGCGGTATCGGGAACCTGTACA 120
het-E-5         GCATCGGGCTCGGGCGACAAGACCATCAAGATCTGGGATACGGCATCGGGAACCTGTACA 120
het-E-6         GCATCGGGCTCGGGCGACAAGACCATCAAGATCTGGGATACGGCATCGGGAACCTGTACA 120
het-E-1         GCATCGGGCTCGGACGACAAGACCATCAAGATCTGGGATACGGCATCGGGAACCGGCACA 120
het-E-7         GCATCGGGCTCGGACGACAAGACCATCAAGATCTGGGATACGGCATCGGGAACCTGTACA 120
het-E-11        GCATCGGGCTCGAGCGATAAGACCATCAAGATCTGGGATACGGCATCGGGAACCTGTACA 120
het-E-12        GCATCGGGCTCGAGCGACAACACCATTAAGATTTGGGATACGGCATCGGGGACCTGCACA 120
het-E-9         GCATCGGGCTCGATCGACGGCACCATTAAGATCTGGGATGCGGCATCGGGGACCTGTACA 120
het-E-10        GCATCGGGCTCGATCGACGGCACCATTAAGATCTGGGATGCGGCATCGGGGACCTGTACA 120
                ************  ***    ***** ***** ****** *** ****** *** * ***

het-E-4         CAGACG 126
e6-3            CAGACG 126
het-E-3         CAGACG 126
het-E-2         CAGACG 126
het-E-8         CAGACG 126
het-E-5         CAGACG 126
het-E-6         CAGACG 126
het-E-1         CAGACA 126
het-E-7         CAGACG 126
het-E-11        CAGACG 126
het-E-12        CAGACG 126
het-E-9         CAGACG 126
het-E-10        CAGACG 126
                *****
```

B/

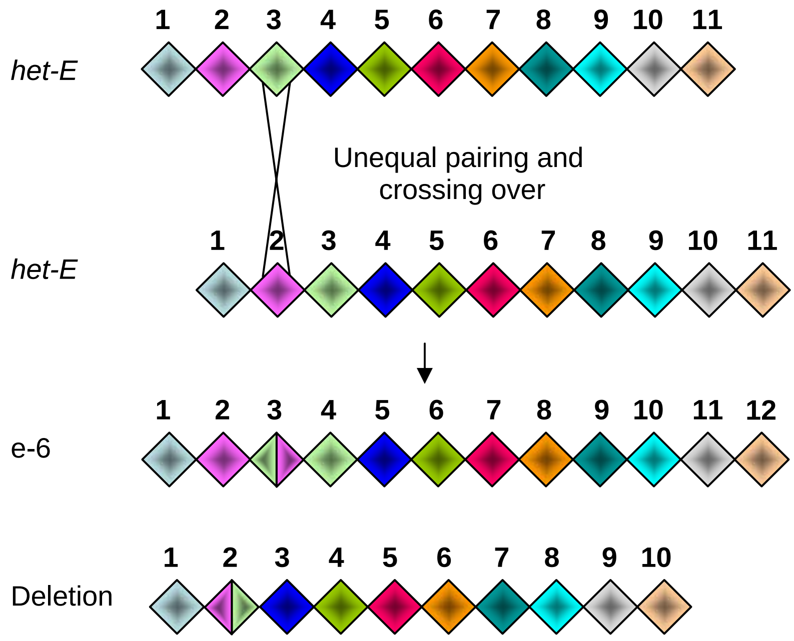
1 2 3 4 5 6 7 8 9 10 11
het-E
Unequal pairing and
crossing over
1 2 3 4 5 6 7 8 9 10 11
het-E
1 2 3 4 5 6 7 8 9 10 11 12
e-6
1 2 3 4 5 6 7 8 9 10
Deletion
