# Supplementary material for: WD-repeat instability and diversification of the Podospora anserina hnwd non-self recognition gene family
Source: BMC Evol Biol. 2010 May 6;10:134. doi: 10.1186/1471-2148-10-134 (PMC2873952; doi:10.1186/1471-2148-10-134)
Supplement: Additional file 8 — Combinations of amino acids found at position 7, 9 25 and 27 of the WD40 repeat units in the hnwd gene family analysed here. [file 1471-2148-10-134-S8.PDF]

Additional file 8:
Combinations of amino acids found at position 7, 9 25 and 27 of the WD40 repeat units in the *HNWD* gene family analysed here. Shaded sequences were already observed previously in (Paoletti et al. 2007).

	Amino acid positions			
WD repeats	7	9	25	27
het-R-1	S	Y	R	V
het-R-2	S	Y	D	V
het-R-4	S	S	R	V
het-R-5	S	S	D	V
het-R-6	S	S	R	I
het-R-7	W	Y	D	V
het-R-9	L	Y	D	V
het-R-10	S	H	D	V
het-R-11	S	S	C	V
het-D$_{\text{het-r hetV}}$-6	W	W	A	S
het-D$_{\text{het-r hetV}}$-5	S	N	T	R
NWD1$_{\text{het-r hetV}}$-2	L	R	M	K
NWD1$_{\text{het-r hetV}}$-6	W	S	G	E
NWD1$_{\text{het-r hetV}}$-7	L	R	G	E
NWDp3$_{\text{het-R het-v}}$-3	L	L	N	G
NWDp3$_{\text{het-R het-v}}$-1	S	R	N	R
NWD2$_{\text{het-r hetV}}$ -1	W	W	G	Y
NWD2$_{\text{het-r hetV}}$ -3	S	L	S	N
NWD2$_{\text{het-r hetV}}$ -5	L	L	H	N
NWDp1$_{\text{het-r hetV}}$ -5	S	W	L	K
NWDp1$_{\text{het-r hetV}}$-7	W	Q	H	K
NWDp1$_{\text{het-r hetV}}$-8	S	Q	H	M
NWDp1$_{\text{het-r hetV}}$-10	X	Q	H	M
NWDp3-4	S	R	N	Q
NWDp3-5	L	L	N	Q
NWDp3-2	S	R	D	G
NWDp3-3	W	R	D	G
NWD2-3	S	R	D	R
NWD2-8	S	R	G	R
NWD2-4	S	W	D	H
NWD2-5	S	W	D	R
NWD2-1	S	G	G	C
NWD2-2	W	W	G	R
hetD-3	W	N	D	H
hetD-4	P	N	D	H
hetD-1	S	N	D	H
hetD-2	W	L	A	S
hetD-5	W	Y	A	S
HNWD1-8	S	N	S	S
HNWD1-9	S	N	G	D
HNWD1-14	F	L	R	K

HNWD1-15	W	M	R	K
HNWD1-1	P	D	R	K
HNWD1-10	S	M	Y	K
HNWD1-6	W	K	D	S
HNWD1-11	W	K	G	K
HNWD1-12	S	H	G	K
HNWD1-13	S	M	N	K
HNWD3-6	T	R	V	E
HNWD3-7	S	R	V	N
HNWD3-3	P	L	V	K
HNWD3-10	S	L	V	K
HNWD3-5	P	W	V	K
HNWD3-9	T	W	V	K
HNWD3-8	P	W	V	E
HNWD3-1	P	R	D	N
HNWD3-2	P	W	D	N
hetE-2	S	W	I	G
NWDp1-1	S	W	L	K
NWDp1-5	S	W	L	M
NWDp1-4	W	Q	H	M
NWDp1-6	W	Q	H	K
NWDp1-2	W	Q	L	M
NWDp1-3	S	Q	V	K
hetE-1	S	L	G	K
NWDp2-4	L	L	H	N
NWDp2-3	L	W	H	N
NWDp2-6	S	W	H	N
NWDp2-2	S	L	S	R
NWDp2-5	S	L	D	R
hetE-3	W	H	S	N
HNWD2-2	S	Y	V	D
HNWD2-3	S	Y	V	C
HNWD2-1	S	Y	G	R
NWD1-4	W	S	D	R
NWD1-3	L	M	D	R
NWD1-7	W	R	G	E
NWD1-8	W	M	G	E
NWD1-5	L	M	G	K
NWD1-6	W	R	H	K
Total amino acids	6	12	14	14
