# Supplementary material for: WD-repeat instability and diversification of the Podospora anserina hnwd non-self recognition gene family
Source: BMC Evol Biol. 2010 May 6;10:134. doi: 10.1186/1471-2148-10-134 (PMC2873952; doi:10.1186/1471-2148-10-134)
Supplement: Additional file 9 — Nucleic and peptidic sequences of het-R mutant alleles affected in the HET or NACHT domain. [file 1471-2148-10-134-S9.PDF]

Additional file 9: Nucleic and peptidic sequences of *het-R* mutant alleles affected in the HET or NACHT domain. In each sequence the mutation is highlighted in red, the three conserved blocks of the HET domain in yellow, the NACHT domain in blue and the WD domain in red. The intron sequence is written in red.

Mutant RV-A1

ATGCGGCTCCTTGAACGTGACGATACCGGCGACTTCCACCTGAAGGATCTGCCCAGCAACGCGATTCCGCCTTACGCGATACTCTCACACACAT
GGGGCGACGAAGAGGTCCTCTTCAAAGATCTGGTGGATGGTACGGGCAGGAAAAATGCAGGCTATGCTAAGGTTCAGTTTTGCGGAGATCAAGC
CTGGCGCGATGGGCTGAAACACTTCTGGATCGACACATGCTGCATCGACAAGTCAGACGCTGTCGAGCTCCAGCACGCTCTCAACTCCATGTTC
CAGTGGTATCGTAACGCCACCAAATGCTATGTCTATCTCACAGACGTCTCAACCCGCAAGTGGGACGCCGACGGCAACTCTGGTTGGGAACTGG
CTTTTCGGACATGCAGATGGTTTACCCGAGGATGGACTCTGCAGGAGCTTATTGCTCCAACGATCGTTGAATTCTTTTCTAAGGAGTGCGAGCG
TTTGGGGGATAAAAAGTCCTTGGAACGAGAAATCCACGATATAACCGGAATTCCTCTAAAAGCTCTTCAGGGAAGACCTCTATCCGATTTCAGT
ATTGCCGAACGAATGGCGTGGATAGAGAAGCGCGATACAAAATTTGAGGAAGATAAGGCTTACTCGCTATTCGGTATCTTTGACGTGCACATAC
CGGTTATTTACGGCGAAGGAAAACAGAAGGCATTGAAGCGGCTGCGAGACAAGATCCGCGAGGATTATCTCTGTTTAGCAAAGCTGTGGTCCGC
TGACCCGCGAGACCCGCACCACGAGAAGGAGCGCATCGAGCTAGCAAAGGGTGGTCTGCTGGCTGATGCTTACCGCTGGGTCTTTTGA

MRLLERDDTGDFHLKDLPSNAIPPYAILSHTWGDEEVLFKDLVDGTGRKNAGYAKVQFCGDQAWRDGLKHFWIDTCCIDKSDAVELQHALNSMF
QWYRNATKCYVYLTDVSTRKWDADGNSGWELAFRTCRWFTRGWTLQELIAPTIVEFFSKECERLGDKKSLEREIHDITGIPLKALQGRPLSDFS
IAERMAWIEKRDTKFEEDKAYSLFGIFDVHIPVIYGEGKQKALKRLRDKIREDYLCLAKLWSADPRDPHHEKERIELAKGGLLADAYRWVF*

Mutant RV-E5

ATGCGGCTCCTTGAACGTGACGATACCGGCGACTTCCACCTGAAGGATCTGCCCAGCAACGCGATTCCGCCTTACGCGATACTCTCACACACAT
GGGGCGACGAAGAGGTCCTCTTCAAAGATCTGGTGGATGGTACGGGCAGGAAAAATGCAGGCTATGCTAAGGTTCAGTTTTGCGGAGATCAAGC
CTGGCGCGATGGGCTGAAACACTTCTGGATCGACACATGCTGCATCGACAAGTCAGACGCTGTCGAGCTCCAGCACGCTCTCAACTCCATGTTC
CAGTGGTATCGTAACGCCACCAAATGCTATGTCTATCTCACAGACGTCTCAACCCGCAAGTGGGACGCCGACGGCAACTCTGGTTGGGAACTGG
CTTTTCGGACATGCAGATGGTTTACCCGAGGATGGACTCTGCAGGAGCTTATTGCTCCAACGATCGTTGAATTCTTTTCTAAGGAGTGCGAGCG
TTTGGGGGATAAAAAGTCCTTGGAACGAGAAATCCACGATATAACCGGAATTCCTCTAAAAGCTCTTCAGGGAAGACCTCTATCCGATTTCAGT
ATTGCCGAACGAATGGCGTGGATAGAGAAGCGCGATACAAAATTTGAGGAAGATAAGGCTTACTCGCTATTCGGTATCTTTGACGTGCACATAC
CGGTTATTTACGGCGAAGGAAAACAGAAGGCATTGAAGCGGCTGCGAGACAAGATCCGCGAGGATTATCTCTGTTTAGCAAAGCTGTGGTCCGC
TGACCCGCGAGACCCGCACCACGAGAAGGAGCGCATCGAGCTAGCAAAGGGTGGTCTGCTGGCTGATGCTTACCGCTGGGTCTTTGACAACCCC
GAATTTCGGCAATGGCGCCATAACTCAGAGAATCGACTTCTCTGGATCAAGGGCGACCCTGGCAAAGGCAAGACCATGTTGCTCTGCGGCATCA
TCGACGAGCTAGAGCAACCCATCACTGCTAGTGGTGGCAACTTGGCGTATTTCTTTTGCCAAGCTACCGACTCGCGCATCAATAGCGCAATTGC
CGTGCTACGGGGGTTGATTTACCTCCTTGCTCGCCGACAACCGGGTCTCCTCTTATATCTGCCCGAGAATACGTACGCTTCCGACGACGCAATG
GCATGGGTCGTTCTATCGAAGGTTTTACGGCGTATGCTAGAAGATCCAGACTTGAAGGAAACCTACCTGGTCATTGATGCCCTTGACGAATGCG
TTATCGACCTACCCAAACTTTTGGATTTCGTCGTCATTTCACCAGGACGCGTCAAGTGGCTCTTATCAAGTCGGAACGAGGTTCTTATTGAGGA
GAAGCTGAAACCTGATGTTGGGCGGACGAGACTTAGTCTCGAGTTGAAAGCGAATGCGATGCAAGTGTCTCACGCCATCGATGCGTATATCGAC
GGCAAGCTATCAGGTCTCGCATCACTTCAAGACGACACGTCGTTGAAAAATCAAGTGCGGGATATTTTGCACCAAAAGGCAAACGGCACGTTCC
TCTGGGTTGCTCTCGTTATACAAGAGCTCAGTGAGGATGATGTTGAGAGCTGGCATGCTCTTCAAATCGTCGAAGAAGTGCCGTCGGGCCTAGA
TAGAATGTACGATCGCATGTTGAATGAAATCAGTCGGTACAAAAGGGACTCGGAATTTTGCCGGCGCATACTCTTGGTGGCCACGGTTGCATAC
CGCCCACTCTACTTGGACGAAATAGGCAGCTTATCTGAATTACCGGAGCAGATTGTGGGATCAACAGAGAATATTAGGAAAGTCGTGGCTAAGT
GCGGATCGTTTCTCACCATCCGAGAAGACAAGATCTACCTTATCCATCAGTCAGCCAAGGACTACTTAAGCGCATCCGCCTTGATCTTCCCCCA
TGGCGCACCAATTGCCCACCGGGATATATGCGCTCGATCGCTAGAGCTTATGTTACAGAAGTTAAGACGCGACATGTACGGCTTAGTTACCCCG
GGATTTCCTATCGACCAGGCCCGCACGCCAGAACCGGACCCTCTTGTGACAATACGGTATTCGTGCGTCTTCTGGGTTGACCACCTCCGCGATT
CGATTACTGATAAAGACACGCCACAACGCAACACACTGGATGCGGTCCAGACGTTCCTTGAACTGAAGTATCTTTACTGGCTCGAAGCTCTTAG
TCTACTCCGAGCTATATCGGAGGTGTCATTGCCATAA

MRLLERDDTGDFHLKDLPSNAIPPYAILSHTWGDEEVLFKDLVDGTGRKNAGYAKVQFCGDQAWRDGLKHFWIDTCCIDKSDAVELQHALNSMF
QWYRNATKCYVYLTDVSTRKWDADGNSGWELAFRTCRWFTRGWTLQELIAPTIVEFFSKECERLGDKKSLEREIHDITGIPLKALQGRPLSDFS
IAERMAWIEKRDTKFEEDKAYSLFGIFDVHIPVIYGEGKQKALKRLRDKIREDYLCLAKLWSADPRDPHHEKERIELAKGGLLADAYRWVFDNP
EFRQWRHNSENRLLWIKGDPGKGKTMLLCGIIDELEQPITASGGNLAYFFCQATDSRINSAIAVLRGLIYLLARRQPGLLLYLPENTYASDDAM
AWVVLSKVLRRMLEDPDLKETYLVIDALDECVIDLPKLLDFVVISPGRVKWLLSSRNEVLIEEKLKPDVGRTRLSLELKANAMQVSHAIDAYID
GKLSGLASLQDDTSLKNQVRDILHQKANGTFLWVALVIQELSEDDVESWHALQIVEEVPSGLDRMYDRMLNEISRYKRDSEFCRRILLVATVAY
RPLYLDEIGSLSELPEQIVGSTENIRKVVAKCGSFLTIREDKIYLIHQSAKDYLSASALIFPHGAPIAHRDICARSLELMLQKLRRDMYGLVTP
GFPIDQARTPEPDPLVTIRYSCVFWVDHLRDSITDKDTPQRNTLDAVQTFLELKYLYWLEALSLLRAISEVSLP*

Mutant RV-J4

ATGCGGCTCCTTGAACGTGACGATACCGGCGACTTCCACCTGAAGGATCTGCCCAGCAACGCGATGCCGCCTTACGCGATACTCTCACACACAT
GGGGCGACGAAGAGGTCCTCTTCAAAGATCTGGTGGATGGTACGGGCAGGAAAAATGCAGGCTATGCTAAGGTTCAGTTTTGCGGAGATCAAGC
CTGGCGCGATGGGCTGAAACACTTCTGGATCGACACATGCTGCATCGACAAGTCAGACGCTGTCGAGCTCCAGCACGCTCTCAACTCCATGTTC
CAGTGGTATCGTAACGCCACCAAATGCTATGTCTATCTCACAGACGTCTCAACCCGCAAGTGGGACGCCGACGGCAACTCTGGTTGGGAACTGG
CTTTTCGGACATGCAGATGGTTTACCCGAGGATGGACTCTGCAGGAGCTTATTGCTCCAACGATCGTTGAATTCTTTTCTAAGGAGTGCGAGCG
TTTGGGGGATAAAAAGTCCTTGGAACGAGAAATCCACGATATAACCGGAATTCCTCTAAAAGCTCTTCAGGGAAGACCTCTATCCGATTTCAGT

ATTGCCGAACGAATGGCGTGGATAGAGAAGCGCGATACAAAATTTGAGGAAGATAAGGCTTACTCGCTATTCGGTATCTTTGACGTGCACATAC
CGGTTATTTACGGCGAAGGAAAACAGAAGGCATTGAAGCGGCTGCGAGACAAGATCCGCGAGGATTATCTCTGTTTAGCAAAGCTGTGGTCCGC
TGACCCGCGAGACCCGCACCACGAGAAGGAGCGCATCGAGCTAGCAAAGGGTGGTCTGCTGGCTGATGCTTACCGCTGGGTCTTTGACAACCCC
GAATTTCGGCAATGGCGCCATAACTCAGAGAATCGACTTCTCTGGATCAAGGGCGACCCTGGCAAAGGCAAGACCATGTTGCTCTGCGGCATCA
TCGACGAGCTAGAGCAACCCATCACTGCTAGTGGTGGCAACTTGGCGTATTTCTTTTGCCAAGCTACCGACTCGCGCATCAATAGCGCAATTGC
CGTGCTACGGGGGTTGATTTACCTCCTTGCTCGCCGACAACCGGGTCTCCTCTTATATCTGCCCGAGAATACGTACGCTTCCGACGACGCAATG
GCATGGGTCGTTCTATCGAAGGTTTTACGGCGTATGCTAGAAGATCCAGACTTGAAGGAAACCTACCTGGTCATTGATGCCCTTGACGAATGCG
TTATCGACCTACCCAAACTTTTGGATTTCGTCGTCATTTCACCAGGACGCGTCAAGTGGCTCTTATCAAGTCGGAACGAGGTTCTTATTGAGGA
GAAGCTGAAACCTGATGTTGGGCGGACGAGACTTAGTCTCGAGTTGAAAGCGAATGCGATGCAAGTGTCTCACGCCATCGATGCGTATATCGAC
GGCAAGCTATCAGGTCTCGCATCACTTCAAGACGACACGTCGTTGAAAAATCAAGTGCGGGATATTTTGCACCAAAAGGCAAACGGCACGTTCC
TCTGGGTTGCTCTCGTTATACAAGAGCTCAGTGAGGATGATGTTGAGAGCTGGCATGCTCTTCAAATCGTCGAAGAAGTGCCGTCGGGCCTAGA
TAGAATGTACGATCGCATGTTGAATGAAATCAGTCGGTACAAAAGGGACTCGGAATTTTGCCGGCGCATACTCTTGGTGGCCACGGTTGCATAC
CGCCCACTCAACTTGGACGAAATAGGCAGCTTATCTGAATTACCGGAGCAGATTGTGGGATCAACAGAGAATATTAGGAAAGTCGTGGCTAAGT
GCGGATCGTTTCTCACCATCCGAGAAGACAAGATCTACCTTATCCATCAGTCAGCCAAGGACTACTTAAGCGCATCCGCCTTGATCTTCCCCCA
TGGCGCACCAATTGCCCACCGGGATATATGCGCTCGATCGCTAGAGCTTATGTTACAGAAGTTAAGACGCGACATGTACGGCTTAGTTACCCCG
GGATTTCCTATCGACCAGGCCCGCACGCCAGAACCGGACCCTCTTGTGACAATACGGTATTCGTGCGTCTTCTGGGTTGACCACCTCCGCGATT
CGATTACTGATAAAGACACGCCACAACGCAACACACTGGATGCGGTCCAGACGTTCCTTGAACTGAAGTATCTTTACTGGCTCGAAGCTCTTAG
TCTACTCCGAGCTATATCGGAGGGTGTCATTGCCATAAGAAAGCTTGAGGGCCTACTAGTAAGTATAGCTTACTAAGACGAGACAGCCATACTA
ACAGAGATACAGGGGCGAACTCATCAAAGGCAGCTAACAACTTTTATCCGGGATGCGCACCGGTTCGCTCTCTCCAACAGATGGATAATCGAGC
AAGCCCCTCTTCAGGCGTATACATCAGCCCTCGTATTTGCACCGGTTGGCAGTCTGGTAAAGAAGAGATTTAAGACGGAAGAACCTAGCTGGAT
CAGTACAAAGCCAGTAGTAGAAACGGACTGGAATGCATGCCTTCAGACGCTCGAAGGCCATAATGGCTCGGTTTACTCGGTCGCCTTTTCGGCG
GATGGCCAGCGGCTCGCATCCGGTGCAGGCGACCGTACCGTCAAGATCTGGGATCCCGCCTCGGGACAATGCTTCCAGACGCTCGAAGGCCATA
ATGGCTCGGTGTACTCGGTCGCCTTTTCGCCGGATGGCCAGCGGCTCGCATCTGGTGCAGTCGACGATACCGTCAAAATCTGGGATCCCGCCTC
GGGACAATGCCTCCAGACGCTCGAAGGCCATAATGGCTCGGTTTACTCGGTCGCCTTTTCGGCGGATGGCCAGCGGCTCGCATCCGGTGCAGGC
GACGATACCGTCAAGATCTGGGATCCCGCCTCGGGACAATGCCTCCAGACGCTCGAAGGCCATAGGGGCTCGGTTTCCTCGGTCGCCTTTTCGG
CGGATGGCCAGCGGCTCGCATCCGGTGCAGTCGACCGTACCGTCAAGATCTGGGATCCCGCCTCGGGACAATGCCTCCAGACGCTCGAAGGCCA
TACGGGCTCGGTTTCCTCGGTCGCCTTTTCGCCGGATGGCCAGCGGTTCGCATCTGGTGTAGTCGACGATACCGTCAAGATCTGGGATCCCGCC
TCGGGACAATGCCTCCAGACGCTCGAAGGCCATAGGGGCTCGGTTTCCTCGGTCGCCTTTTCGCCGGATGGCCAGCGGTTCGCATCCGGTGCAG
GCGACCGTACCATCAAGATCTGGGATCCCGCCTCGGGACAATGCCTCCAGACGCTCGAAGGCCATAGGGGCTGGGTTTACTCGGTCGCCTTTTC
GGCGGATGGCCAGCGGTTCGCATCCGGTGCAGGCGACGATACCGTCAAAATCTGGGATCCCGCCTCGGGACAATGCCTCCAGACGCTTGAAAGC
CATAATGGCTCGGTTTCCTCGGTCGCCTTTTCGCCGGATGGCCAGCGGCTCGCATCTGGTGCAGACGACGATACCGTCAAAATCTGGGATCCCG
CCTCGGGACAATGCCTCCAGACACTCGAAGGCCATAAGGGCTTGGTTTACTCGGTTACCTTTTCGGCGGATGGCCAGCGGCTCGCATCTGGTGC
AGGCGACGATACCGTCAAAATCTGGGATCCCGCCTCGGGACAATGCCTCCAGACGCTCGAAGGCCATAGGGGCTCGGTTCACTCGGTCGCCTTT
TCGCCGGATGGCCAGCGGTTCGCATCTGGTGCAGTCGACGATACCGTCAAGATCTGGGATCCCGCCTCGGGACAATGCCTCCAGACGCTCGAAG
GCCATAATGGCTCGGTTTCCTCGGTCGCCTTTTCGGCGGATGGCCAGCGGCTCGCATCTGGTGCAGTCGACTGTACCGTCAAAATCTGGGATCC
CGCCTCGGGACAATGCCTCCAGACGCTCGAAGGCTATAGGAGTTCGGTTTCCTCGGTCGCCTTTTTGGCAGATAATCAGGGGGCGCATGGGTAT
AGGTTGGGCCAAGACACGACCTGGGTCATTTGTAACGGCCAGAATGTGCTATGGTTACCACCTGAATACCGCCCAACCTGCTCTGCAATCCAGG
GGCGGATAGTAGCTATTGGCTGTTCATCAGGGCGAGTTTTTACTATTGGCTTCTCAAGAGACAAATAA

MRLLERDDTGDFHLKDLPSNAMPPYAILSHTWGDEEVLFKDLVDGTGRKNAGYAKVQFCGDQAWRDGLKHFWIDTCCIDKSDAVELQHALNSMFQ
WYRNATKCYVYLTDVSTRKWDADGNSGWELAFRTCRWFTRGWTLQELIAPTIVEFFSKECERLGDKKSLEREIHDITGIPLKALQGRPLSDFSIA
ERMAWIEKRDTKFEEDKAYSLFGIFDVHIPVIYGEGKQKALKRLRDKIREDYLCLAKLWSADPRDPHHEKERIELAKGGLLADAYRWVFDNPEFR
QWRHNSENRLLWIKGDPGKGKTMLLCGIIDELEQPITASGGNLAYFFCQATDSRINSAIAVLRGLIYLLARRQPGLLLYLPENTYASDDAMAWVV
LSKVLRRMLEDPDLKETYLVIDALDECVIDLPKLLDFVVISPGRVKWLLSSRNEVLIEEKLKPDVGRTRLSLELKANAMQVSHAIDAYIDGKLSG
LASLQDDTSLKNQVRDILHQKANGTFLWVALVIQELSEDDVESWHALQIVEEVPSGLDRMYDRMLNEISRYKRDSEFCRRILLVATVAYRPLYLD
EIGSLSELPEQIVGSTENIRKVVAKCGSFLTIREDKIYLIHQSAKDYLSASALIFPHGAPIAHRDICARSLELMLQKLRRDMYGLVTPGFPIDQA
RTPEPDPLVTIRYSCVFWVDHLRDSITDKDTPQRNTLDAVQTFLELKYLYWLEALSLLRAISEGVIAIRKLEGLLGRTHQRQLTTFIRDAHRFAL
SNRWIIEQAPLQAYTSALVFAPVGSLVKKRFKTEEPSWISTKPVVETDWNACLQTLEGHNGSVYSVAFSADGQRLASGAGDRTVKIWDPASGQCF
QTLEGHNGSVYSVAFSPDGQRLASGAVDDTVKIWDPASGQCLQTLEGHNGSVYSVAFSADGQRLASGAGDDTVKIWDPASGQCLQTLEGHRGSVS
SVAFSADGQRLASGAVDRTVKIWDPASGQCLQTLEGHTGSVSSVAFSPDGQRFASGVVDDTVKIWDPASGQCLQTLEGHRGSVSSVAFSPDGQRF
ASGAGDRTIKIWDPASGQCLQTLEGHRGWVYSVAFSADGQRFASGAGDDTVKIWDPASGQCLQTLESHNGSVSSVAFSPDGQRLASGADDDTVKI
WDPASGQCLQTLEGHKGLVYSVTFSADGQRLASGAGDDTVKIWDPASGQCLQTLEGHRGSVHSVAFSPDGQRFASGAVDDTVKIWDPASGQCLQT
LEGHNGSVSSVAFSADGQRLASGAVDCTVKIWDPASGQCLQTLEGYRSSVSSVAFLADNQGAHGYGLGQDTTWVICNGQNVLWLPPEYRPTCSAI
QGRIVAIGCSSGRVFTIGFSRDK

Mutant RV-M8

ATGCGGCTCCTTGAACGTGACGATACCGGCGACTTCCACCTGAAGGATCTGCCCAGCAACGCGATTCCGCCTTACGCGATACTCTCACACACAT
GGGGCGACGAAGAGGTCCTCTTCAAAGATCTGGTGGATGGTACGGGCAGGAAAAATGCAGGCTATGCTAAGGTTCAGTTTTGCGGAGATCAAGC
CTGGCGCGATGGGCTGAAACACTTCTGGATCGACACATGCTGCATCGACAAGTCAGACGCTGTCGAGCTCCAGCACGCTCTCAACTCCATGTTC
CAGTGGTATCGTAACGCCACCAAATGCTATGTCTATCTCACAGACGTCTCAACCCGCAAGTGGGACGCCGACGGCAACTCTGGTTGGGAACTGG
CTTTTCGGACATGCAGATGGTTTACCCGAGGATGGACTCTGCAGGAGCTTATTGCTCCAACGATCGTTGAATTCTTTTCTAAGGAGTGCGAGCG
TTTGGGGGATAAAAAGTCCTTGGAACGAGAAATCCACGATATAACCGGAATTCCTCTAAAAGCTCTTCAGGGAAGACCTCTATCCGATTTCAGT
ATTGCCGAACGAATGGCGTGGATAGAGAAGCGCGATACAAAATTTGAGGAAGATAAGGCTTACTCGCTATTCGGTATCTTTGACGTGCACATAC
CGGTTATTTACGGCGAAGGAAAACAGAAGGCATTGAAGCGGCTGCGAGACAAGATCCGCGAGGATTATCTCTGTTTAGCAAAGCTGTGGTCCGC
TGACCCGCGAGACCCGCACCACGAGAAGGAGCGCATCGAGCTAGCAAAGGGTGGTCTGCTGGCTGATGCTTACCGCTGGGTCTTTGACAACCCC
GAATTTCGGCAATGGCGCCATAACTCAGAGAATCGACTTCTCTGGATCAAGGGCGACCCTGGCAAAGGCAAGACCATGTTGCTCTGCGGCATCA
TCGACGAGCTAGAGCAACCCATCACTGCTAGTGGTGGCAACTTGGCGTATTTCTTTTGCCAAGCTACCGACTCGCGCATCAATAGCGCAATTGC
CGTGCTACGGGGGTTGATTTACCTCCTTGCTCGCCGACAACCGGGTCTCCTCTTATATCTGCCCGAGAATACGTACGCTTCCGACGACGCAATG
GCATGGGTCGTTCTATCGAAGGTTTTACGGCGTATGCTAGAAGATCCAGACTTGAAGGAAACCTACCTGGTCATTGATGCCCTTGACGAATGCG
TTATCGACCTACCCAAACTTTTGGATTTCGTCGTCATTTCACCAGGACGCGTCAAGTGGCTCTTATCAAGTCGGAACGAGGTTCTTATTGAGGA
GAAGCTGAAACCTGATGTTGGGCGGACGAGACTTAGTCTCGAGTTGAAAGCGAATGCGATGCAAGTGTCTCACGCCATCGATGCGTATATCGAC

GGCAAGCTATCAGGTCTCGCATCACTTCAAGACGACACGTCGTTGAAAAATCAAGTGCGGGATATTTTGCACCAAAAGGCAAACGGCACGTTCC
TCTGGGTTGCTCTCGTTATACAAGAGCTCAGTGAGGATGATGTTGAGAGCTGGCATGCTCTTCAAATCGTCGAAGAAGTGCCGTCGGGCCTAGA
TAGAATGTACGATCGCATGTTGAATGAAATCAGTCGGTACAAAAGGGACTCGGAATTTTGCCGGCGCATACTCTTGGTGGCCACGGTTGCATAC
CGCCCACTCTACTTGGACGAAATAGGCAGCTTATCTGAATTACCGGAGCAGATTGTGGGATCAACAGAGAATATTAGGAAAGTCGTGGCTAAGT
GCGGATCGTTTCTCACCATCCGAGAAGACAAGATCTACCTTATCCATCAGTCAGCCAAGGACTACTTAAGCGCATCCGCCTTGATCTTCCCCCA
TGGCGCACCAATTGCCCACCGGGATATATGCGCTCGATCGCTAGAGCTTATGTTACAGAAGTTAAGACGCGACATGTACGGCTTAGTTACCCCG
GGATTTCCTATCGACCAGGCCCGCACGCCAGAACCGGACCCTCTTGTGACAATACGGTATTCGTGCGTCTTCTGGGTTGACCACCTCCGCGATT
CGATACTGATAAAGACACGCCACAACGCAACACACTGGATGCGGTCCAGACGTTCCTTGAACTGA

MRLLERDDTGDFHLKDLPSNAIPPYAILSHTWGDEEVLFKDLVDGTGRKNAGYAKVQFCGDQAWRDGLKHFWIDTCCIDKSDAVELQHALNSMF
QWYRNATKCYVYLTDVSTRKWDADGNSGWELAFRTCRWFTRGWTLQELIAPTIVEFFSKECERLGDKKSLEREIHDITGIPLKALQGRPLSDFS
IAERMAWIEKRDTKFEEDKAYSLFGIFDVHIPVIYGEGKQKALKRLRDKIREDYLCLAKLWSADPRDPHHEKERIELAKGGLLADAYRWVFDNP
EFRQWRHNSENRLLWIKGDPGKGKTMLLCGIIDELEQPITASGGNLAYFFCQATDSRINSAIAVLRGLIYLLARRQPGLLLYLPENTYASDDAM
AWVVLSKVLRRMLEDPDLKETYLVIDALDECVIDLPKLLDFVVISPGRVKWLLSSRNEVLIEEKLKPDVGRTRLSLELKANAMQVSHAIDAYID
GKLSGLASLQDDTSLKNQVRDILHQKANGTFLWVALVIQELSEDDVESWHALQIVEEVPSGLDRMYDRMLNEISRYKRDSEFCRRILLVATVAY
RPLYLDEIGSLSELPEQIVGSTENIRKVVAKCGSFLTIREDKIYLIHQSAKDYLSASALIFPHGAPIAHRDICARSLELMLQKLRRDMYGLVTP
GFPIDQARTPEPDPLVTIRYSCVFWVDHLRDSILIKTRHNATHWMRSRRSLN*

Mutant RV-M12

ATGCGGCTCCTTGAACGTGACGATACCGGCGACTTCCACCTGAAGGATCTGCCCAGCAACGCGATTCCGCCTTACGCGATACTCTCACACACAT
GGGGCGACGAAGAGGTCCTCTTCAAAGATCTGGTGGATGGTACGGGCAGGAAAAATGCAGGCTATGCTAAGGTTCAGTTTTGCGGAGATCAAGC
CTGGCGCGATGGGCTGAAACACTTCTGGATCGACACATGCTGCATCGACAAGTCAGACGCTGTCGAGCTCCAGCACGCTCTCAACTCCATGTTC
CAGTGGTATCGTAACGCCACCAAATGCTATGTCTATCTCACAGACGTCTCAACCCGCAAGTGGGACGCCGACGGCAACTCTGGTTGGGAACTGG
CTTTTCGGACATGCAGATGGTTTACCCGAGGATGGACTCTGCAGGAGCTTATTGCTCCAACGATCGTTGAATTCTTTTCTAAGGAGTGCGAGCG
TTTGGGGGATAAAAAGTCCTTGGAACGAGAAATCCACGATATAACCGGAATTCCTCTAAAAGCTCTTCAGGGAAGACCTCTATCCGATTTCAGT
ATTGCCGAACGAATGGCGTGGATAGAGAAGCGCGATACAAAATTTGAGGAAGATAAGGCTTACTCGCTATTCGGTATCTTTGACGTGCACATAC
CGGTTATTTACGGCGAAGGAAAACAGAAGGCATTGAAGCGGCTGCGAGACAAGATCCGCGAGGATTATCTCTGTTTAGCAAAGCTGTGGTCCGC
TGACCCGCGAGACCCGCACCACGAGAAGGAGCGCATCGAGCTAGCAAAGGGTGGTCTGCTGGCTGATGCTTACCGCTGGGTCTTTGACAACCCC
GAATTTCGGCAATGGCGCCATAACTCAGAGAATCGACTTCTCTGGATCAAGGGCGACCCTGGCAAAGGCAAGACCATGTTGCTCTGCGGCATCA
TCGACGAGCTAGAGCAACCCATCACTGCTAGTGGTGGCAACTTGGCGTATTTCTTTTGCCAAGCTACCGACTCGCGCATCAATAGCGCAATTGC
CGTGCTACGGGGGTTGATTTACCTCCTTGCTCGCCGACAACCGGGTCTCCTCTTATATCTGCCCGAGAATACGTACGCTTCCGACGACGCAATG
GCATGGGTCGTTCTATCGAAGGTTTTACGGCGTATGCTAGAAGATCCAGACTTGAAGGAAACCTACCTGGTCATTGATGCCCTTGACGAATGCG
TTATCGACCTACCCAAACTTTTGGATTTCGTCGTCATTTCACCAGGACGCGTCAAGTGGCTCTTATCAAGTCGGAACGAGGTTCTTATTGAGGA
GAAGCTGAAACCTGATGTTGGGCGGACGAGACTTAGTCTCGAGTTGAAAGCGAATGCGATGCAAGTGTCTCACGCCATCGATGCGTATATCGAC
GGCAAGCTATCAGGTCTCGCATCACTTCAAGACGACACGTCGTTGAAAAATCAAGTGCGGGATATTTTGCACCAAAAGGCAAACGGCACGTTCC
TCTGGGTTGCTCTCGTTATACAAGAGCTCAGTGAGGATGATGTTGAGAGCTGGCATGCTCTTCAAATCGTCGAAGAAGTGCCGTCGGGCCTAGA
TAGAATGTACGATCGCATGTTGAATGAAATCAGTCGGTACAAAAGGGACTCGGAATTTTGCCGGCGCATACTCTTGGTGGCCACGGTTGCATAC
CGCCCACTCAACTTGGACGAAATAGGCAGCTTATCTGAATTACCGGAGCAGATTGTGGGATCAACAGAGAATATTAGGAAAGTCGTGGCTAAGT
GCGGATCGTTTCTCACCATCCGAGAAGACAAGATCTACCTTATCCATCAGTCAGCCAAGGACTACTTAAGCGCATCCGCCTTGATCTTCCCCCA
TGGCGCACCAATTGCCCACCGGGATATATGCGCTCGATCGCTAGAGCTTATGTTACAGAAGTTAAGACGCGACATGTACGGCTTAGTTACCCCG
GGATTTCCTATCGACCAGGCCCGCACGCCAGAACCGGACCCTCTTGTGACAATACGGTATTCGTGCGTCTTCTGGGTTGACCACCTCCGCGATT
CGATTACTGATAAAGACACGCCACAACGCAACACACTGGATGCGGTCCAGACGTTCCTTGAACTGAAGTATCTTTACTGGCTCGAAGCTCTTAG
TCTACTCCGAGCTATATCGGAGGGTGTCATTGCCATAAGAAAGCTTGAGGGCCTACTAGTAAGTATAGCTTACTAAGACGAGACAGCCATACTA
ACAGAGATACAGGGGCGAACTCATCAAAGGCAGCTAACAACTTTTATCCGGGATGCGCACCGGTTCGCTCTCTCCAACAGATGGATAATCGAGC
AAGCCCCTCTTCAGGCGTATACATCAGCCCTCGTATTTGCACCGGTTGGCAGTCTGGTAAAGAAGAGATTTAAGACGGAAGAACCTAGCTGGAT
CAGTACAAAGCCAGTAGTAGAAACGGACTGGAATGCATGCCTTCAGACGCTCGAAGGCCATAATGGCTCGGTTTACTCGGTCGCCTTTTCGGCG
GATGGCCAGCGGCTCGCATCCGGTGCAGGCGACCGTACCGTCAAGATCTGGGATCCCGCCTCGGGACAATGCTTCCAGACGCTCGAAGGCCATA
ATGGCTCGGTGTACTCGGTCGCCTTTTCGCCGGATGGCCAGCGGCTCGCATCTGGTGCAGTCGACGATACCGTCAAAATCTGGGATCCCGCCTC
GGGACAATGCCTCCAGACGCTCGAAGGCCATAATGGCTCGGTTTACTCGGTCGCCTTTTCGGCGGATGGCCAGCGGCTCGCATCCGGTGCAGGC
GACGATACCGTCAAGATCTGGGATCCCGCCTCGGGACAATGCCTCCAGACGCTCGAAGGCCATAGGGGCTCGGTTTCCTCGGTCGCCTTTTCGG
CGGATGGCCAGCGGCTCGCATCCGGTGCAGTCGACCGTACCGTCAAGATCTGGGATCCCGCCTCGGGACAATGCCTCCAGACGCTCGAAGGCCA
TACGGGCTCGGTTTCCTCGGTCGCCTTTTCGCCGGATGGCCAGCGGTTCGCATCTGGTGTAGTCGACGATACCGTCAAGATCTGGGATCCCGCC
TCGGGACAATGCCTCCAGACGCTCGAAGGCCATAGGGGCTCGGTTTCCTCGGTCGCCTTTTCGCCGGATGGCCAGCGGTTCGCATCCGGTGCAG
GCGACCGTACCATCAAGATCTGGGATCCCGCCTCGGGACAATGCCTCCAGACGCTCGAAGGCCATAGGGGCTGGGTTTACTCGGTCGCCTTTTC
GGCGGATGGCCAGCGGTTCGCATCCGGTGCAGGCGACGATACCGTCAAAATCTGGGATCCCGCCTCGGGACAATGCCTCCAGACGCTTGAAAGC
CATAATGGCTCGGTTTCCTCGGTCGCCTTTTCGCCGGATGGCCAGCGGCTCGCATCTGGTGCAGACGACGATACCGTCAAAATCTGGGATCCCG
CCTCGGGACAATGCCTCCAGACACTCGAAGGCCATAAGGGCTTGGTTTACTCGGTTACCTTTTCGGCGGATGGCCAGCGGCTCGCATCTGGTGC
AGGCGACGATACCGTCAAAATCTGGGATCCCGCCTCGGGACAATGCCTCCAGACGCTCGAAGGCCATAGGGGCTCGGTTCACTCGGTCGCCTTT
TCGCCGGATGGCCAGCGGTTCGCATCTGGTGCAGTCGACGATACCGTCAAGATCTGGGATCCCGCCTCGGGACAATGCCTCCAGACGCTCGAAG
GCCATAATGGCTCGGTTTCCTCGGTCGCCTTTTCGGCGGATGGCCAGCGGCTCGCATCTGGTGCAGTCGACTGTACCGTCAAAATCTGGGATCC
CGCCTCGGGACAATGCCTCCAGACGCTCGAAGGCTATAGGAGTTCGGTTTCCTCGGTCGCCTTTTTGGCAGATAATCAGGGGGCGCATGGGTAT
AGGTTGGGCCAAGACACGACCTGGGTCATTTGTAACGGCCAGAATGTGCTATGGTTACCACCTGAATACCGCCCAACCTGCTCTGCAATCCAGG
GGCGGATAGTAGCTATTGGCTGTTCATCAGGGCGAGTTTTTACTATTGGCTTCTCAAGAGACAAATAA

MRLLERDDTGDFHLKDLPSNAIPPYAILSHTWGDEEVLFKDLVDGTGRKNAGYAKVQFCGDQAWRDGLKHFWIDTCCIDKSDAVELQHALNSMF
QWYRNATKCYVYLTDVSTRKWDADGNSGWELAFRTCRWFTRGWTLQELIAPTIVEFFSKECERLGDKKSLEREIHDITGIPLKALQGRPLSDFS
IAERMAWIEKRDTKFEEDKAYSLFGIFDVHIPVIYGEGKQKALKRLRDKIREDYLCLAKLWSADPRDPHHEKERIELAKGGLLADAYRWVFDNP
EFRQWRHNSENRLLWIKGDPGKGKTMLLCGIIDELEQPITASGGNLAYFFCQATDSRINSAIAVLRGLIYLLARRQPGLLLYLPENTYASDDAM
AWVVLSKVLRRMLEDPDLKETYLVIDALDECVIDLPKLLDFVVISPGRVKWLLSSRNEVLIEEKLKPDVGRTRLSLELKANAMQVSHAIDAYID
GKLSGLASLQDDTSLKNQVRDILHQKANGTFLWVALVIQELSEDDVESWHALQIVEEVPSGLDRMYDRMLNEISRYKRDSEFCRRILLVATVAY

RPLNLDEIGSLSELPEQIVGSTENIRKVVAKCGSFLTIREDKIYLIHQSAKDYLSASALIFPHGAPIAHRDICARSLELMLQKLRRDMYGLVTP
GFPIDQARTPEPDPLVTIRYSCVFWVDHLRDSITDKDTPQRNTLDAVQTFLELKYLYWLEALSLLRAISEGVIAIRKLEGLLGRTHQRQLTTFI
RDAHRFALSNRWIIEQAPLQAYTSALVFAPVGSLVKKRFKTEEPSWISTKPVVETDWNACLQTLEGHNGSVYSVAFSADGQRLASGAGDRTVKI
WDPASGQCFQTLEGHNGSVYSVAFSPDGQRLASGAVDDTVKIWDPASGQCLQTLEGHNGSVYSVAFSADGQRLASGAGDDTVKIWDPASGQCLQ
TLEGHRGSVSSVAFSADGQRLASGAVDRTVKIWDPASGQCLQTLEGHTGSVSSVAFSPDGQRFASGVVDDTVKIWDPASGQCLQTLEGHRGSVS
SVAFSPDGQRFASGAGDRTIKIWDPASGQCLQTLEGHRGWVYSVAFSADGQRFASGAGDDTVKIWDPASGQCLQTLESHNGSVSSVAFSPDGQR
LASGADDDTVKIWDPASGQCLQTLEGHKGLVYSVTFSADGQRLASGAGDDTVKIWDPASGQCLQTLEGHRGSVHSVAFSPDGQRFASGAVDDTV
KIWDPASGQCLQTLEGHNGSVSSVAFSADGQRLASGAVDCTVKIWDPASGQCLQTLEGYRSSVSSVAFLADNQGAHGYGLGQDTTWVICNGQNV
LWLPPEYRPTCSAIQGRIVAIGCSSGRVFTIGFSRDK
