# Supplementary material for: WD-repeat instability and diversification of the Podospora anserina hnwd non-self recognition gene family
Source: BMC Evol Biol. 2010 May 6;10:134. doi: 10.1186/1471-2148-10-134 (PMC2873952; doi:10.1186/1471-2148-10-134)
Supplement: Additional file 10 — List of the accession numbers for the sequences used in this study. [file 1471-2148-10-134-S10.PDF]

Additional file 10:

List of the accession numbers for the sequences used in this study:

Name	Access number	
het-E-1 (WT)	L28125	(Saupe et al. 1995)
het-R	FJ269239	(Chevanne et al. 2009)
het-E (WT)	FJ897789	
CE-A7	FJ897791	[e1]
CE-C6	FJ897796	[e2]
CE-A12	FJ897792	[e3]
CE-A2	FJ897790	[e4]
CE-B1	FJ897793	[e5]
CE-B6	FJ897794	[e6]
CE-B13	FJ897795	[e7]
CE-C8	FJ897797	[e8]
CE-C4	FJ897798	[e9]
RV-D3	FJ897799	[r1]
RV-G12	FJ897801	[r2]
RV-G2	FJ897800	[r3]
RV-G17	FJ897802	[r4]
RV-I4	FJ897803	[r5]
RV-H3	FJ897805	[r6]
RV-L9	FJ897806	[r7]
RV-L12	FJ897807	[r8]
RV-C1	FJ897804	[r9]
RV-M10	FJ897809	[r10]
RV-H9	FJ897808	[r11]
RV-A1	FJ897828	[r12]
RV-E5	FJ897829	[r13]
RV-J4	FJ897830	[r14]
RV-M8	FJ897831	[r15]
RV-M12	FJ897832	[r16]
het-d$_{\text{het-r het-V}}$	FJ897810	
het-d$_{\text{het-R.het-V1}}$	FJ897811	
het-e$_{\text{het-r het-V}}$	FJ897812	
het-e $_{\text{het-R.het-V1}}$	FJ897813	
HNWD1$_{\text{het-r het-V}}$	FJ897814	
HNWD1 $_{\text{het-R.het-V1}}$	FJ897815	
HNWD3$_{\text{het-r het-V}}$	FJ897816	
HNWD3 $_{\text{het-R.het-V1}}$	FJ897817	
NWD1$_{\text{het-r het-V}}$	FJ897818	
NWD1 $_{\text{het-R.het-V1}}$	FJ897819	
NWD2$_{\text{het-r het-V}}$	FJ897820	
NWD2 $_{\text{het-R.het-V1}}$	FJ897821	
NWDp1$_{\text{het-r het-V}}$	FJ897822	
NWDp1 $_{\text{het-R.het-V1}}$	FJ897823	
NWDp2$_{\text{het-r het-V}}$	FJ897824	
NWDp2 $_{\text{het-R.het-V1}}$	FJ897825	
NWDp3$_{\text{het-r het-V}}$	FJ897826	
NWDp3 $_{\text{het-R.het-V1}}$	FJ897827	

For the members of the *NWD* family other than *het-e* or *het-r*, the strain of origin is indicated in subscript. The names of the mutants correspond to the incompatibility system concerned (RV or CE), the spore from which the mutant is derived (letter) and a number. For easy reading we attributed simplified names to mutants described in the text that are indicated in brackets.
